# Metrological complementarity reveals the Einstein-Podolsky-Rosen paradox

**DOI:** 10.1038/s41467-021-22353-3

**Published:** 2021-04-23

**Authors:** Benjamin Yadin, Matteo Fadel, Manuel Gessner

**Affiliations:** 1 School of Mathematical Sciences and Centre for the Mathematics and Theoretical Physics of Quantum Non-Equilibrium Systems, University of Nottingham, Nottingham, UK; 2 Wolfson College, University of Oxford, Oxford, UK; 3 Department of Physics, University of Basel, Basel, Switzerland; 4 Laboratoire Kastler Brossel, ENS-Université PSL, CNRS, Sorbonne Université, Collège de France, Paris, France

**Keywords:** Quantum information, Quantum metrology

## Abstract

The Einstein-Podolsky-Rosen (EPR) paradox plays a fundamental role in our understanding of quantum mechanics, and is associated with the possibility of predicting the results of non-commuting measurements with a precision that seems to violate the uncertainty principle. This apparent contradiction to complementarity is made possible by nonclassical correlations stronger than entanglement, called steering. Quantum information recognises steering as an essential resource for a number of tasks but, contrary to entanglement, its role for metrology has so far remained unclear. Here, we formulate the EPR paradox in the framework of quantum metrology, showing that it enables the precise estimation of a local phase shift and of its generating observable. Employing a stricter formulation of quantum complementarity, we derive a criterion based on the quantum Fisher information that detects steering in a larger class of states than well-known uncertainty-based criteria. Our result identifies useful steering for quantum-enhanced precision measurements and allows one to uncover steering of non-Gaussian states in state-of-the-art experiments.

## Abstract (web summary)

Steering reflects the ability to predict measurement results on one side of a quantum-correlated system based on measurements on the other side, which can be phrased as a metrology problem. Here, the authors explore this connection, deriving a general steering criterion based on quantum Fisher information.

## Introduction

In their seminal 1935 paper^1^, EPR presented a scenario where the position and momentum of one quantum system (B) can both be predicted with certainty from local measurements of another remote system (A). Based on this apparent violation of the uncertainty principle, in 1989 Reid formulated the first practical criterion for an EPR paradox^2^, which has enabled numerous experimental observations^3^: Steering from A to B is revealed when measurement results of A allow one to predict the measurement results of B with errors that are smaller than the limit imposed by the Heisenberg–Robertson uncertainty relation for B. More generally, an EPR paradox implies the failure of any attempt to describe the correlations between the two systems in terms of classical probability distributions and local quantum states for B, known as local hidden state (LHS) models, as was shown by Wiseman et al.^4^ using the framework of quantum information theory. Aside from its fundamental interest, steering is recognised as an essential resource for quantum information tasks^5^, such as one-sided device-independent quantum key distribution^6,7^ and quantum channel discrimination^8^.

Uncertainty relations describe the complementarity of non-commuting observables, but the complementarity principle applies more generally to notions that are not necessarily associated with an operator. One generalisation^9^ involves the quantum Fisher information (QFI), the central tool for quantifying the precision of quantum parameter estimation^10–13^. Besides its fundamental relevance for quantum-enhanced precision measurements, the QFI is of great interest for the characterisation of quantum many-body systems^14,15^ and gives rise to an efficient and experimentally accessible witness for multipartite entanglement^12,13,16^, but so far, its relation to steering has remained elusive. It has been a long-standing open problem to determine if quantum correlations stronger than entanglement, such as steering or Bell correlations, play a role in metrology^17^.

In this work we formulate a steering condition in terms of the complementarity of a phase shift *θ* and its generating Hamiltonian *H*, using information-theoretic tools from quantum metrology. We express our steering condition in terms of the QFI. The more general phase-generator complementarity principle reproduces the Heisenberg–Robertson uncertainty relation in the special case where the phase is estimated from an observable *M*. Therefore, our metrological criterion is stronger than the uncertainty-based approach and allows us to uncover hidden EPR paradoxes in experimentally relevant scenarios. Our result answers positively the question of whether steering can be a resource in quantum sensing applications.

## Results

### Reid’s criterion for an EPR paradox

**Fig. 1:**
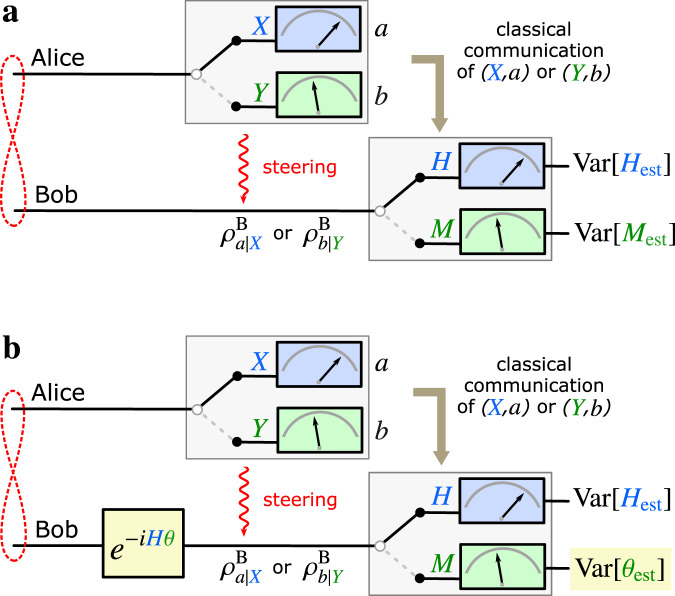
Formulation of the EPR paradox as a metrological task. **a** In the standard EPR scenario, Alice’s measurement setting *X* (*Y*), and result *a* (*b*), leave Bob in the conditional quantum states ${\rho }_{a| X}^{{{{\rm{B}}}}}$ (${\rho }_{b| Y}^{{{{\rm{B}}}}}$). Knowing Alice’s setting and result allows Bob to choose what measurement to perform on his state, and to make a prediction for the result. In an ideal scenario with strong quantum correlations, Alice’s measurement of *X* (*Y*) steers Bob into an eigenstate of his observable *H* (*M*), allowing him to predict the result with certainty. When *H* and *M* do not commute, this seems to contradict the complementarity principle. In practice, an EPR paradox is revealed whenever Bob’s predictions are precise enough to observe an apparent violation of Heisenberg’s uncertainty relation, see Eq. (1). **b** In our formulation of the EPR paradox as a metrological task, a local phase shift *θ* is generated by *H* on Bob’s state. Then, depending on Alice’s measurement setting and result, he decides whether to predict and measure *H* (as before), or to estimate *θ* from the measurement *M*. Here, Bob can choose the observable *M* as a function of Alice’s measurement result. The complementarity between *θ* and its generator *H* seems to be contradicted if the lower bound on their estimation errors, Eq. (3), is violated. This gives a metrological criterion for observing the EPR paradox. Since the metrological complementarity is sharper than the uncertainty-based notion, this approach leads to a tighter criterion to detect steering. Both results coincide in the special case when Bob estimates *θ* only from the observable *M*.

We first recall some basic definitions by considering the following scenario (see Fig. 1a). Alice (A) performs a measurement on her subsystem and communicates her setting *X* and result *a* to Bob (B). Based on this information, Bob uses an estimator *h*_est_(*a*) to predict the result of his subsequent measurement of $H={\sum }_{h}h\left|h\right\rangle \left\langle h\right|$. The average deviation between the prediction and Bob’s actual result *h* is given by ${{{\rm{Var}}}}[{H}_{{{{\rm{est}}}}}]:={\sum }_{a,h}p(a,h| X,H){\left({h}_{{{{\rm{est}}}}}(a)-h\right)}^{2}$, often called the inference variance^3^, where *p*(*a*, *h*∣*X*, *H*) is the joint probability distribution for results *a* and *h*, conditioned on the measurement settings *X* and *H*. The procedure is repeated with different measurement settings *Y* and *M*, and Reid’s criterion^2,3^ for an EPR paradox consists of a violation of the local uncertainty limit

1 $$\begin{array}{l}{{{\rm{Var}}}}[{H}_{{{{\rm{est}}}}}]{{{\rm{Var}}}}[{M}_{{{{\rm{est}}}}}]\ge \frac{| {\langle [H,M]\rangle }_{{\rho }^{{{{\rm{B}}}}}}{| }^{2}}{4}.\end{array}$$

From the perspective of quantum information theory, the condition (1) plays the role of a witness for steering, but it may not always succeed in revealing an EPR paradox.

The most general way to formally model the joint statistics *p*(*a*, *h*∣*X*, *H*) is offered by the formalism of assemblages, *i.e*. functions ${{{\mathcal{A}}}}(a,X)=p(a| X){\rho }_{a| X}^{{{{\rm{B}}}}}$ that map any possible result *a* of Alice’s measurement of *X* to a local probability distribution *p*(*a*∣*X*) and a (normalised) conditional quantum state ${\rho }_{a| X}^{{{{\rm{B}}}}}$ for Bob’s subsystem^18^. This description avoids the need to make assumptions about the nature of Alice’s system, which is key to one-sided device-independent quantum information processing^5–7^. We only impose a no-signalling condition which requires that ${\sum }_{a}{{{\mathcal{A}}}}(a,X)={\rho }^{{{{\rm{B}}}}}$ for all *X*, where *ρ*^B^ is the reduced density matrix of Bob’s system. Based on the assemblage ${{{\mathcal{A}}}}$, the joint statistics are described as $p(a,h| X,H)=p(a| X)\left\langle h\right|{\rho }_{a| X}^{{{{\rm{B}}}}}\left|h\right\rangle$.

The EPR paradox can now be formally defined as an observation that rules out the possibility of modelling an assemblage by a LHS model. In such a model, a classical random variable *λ* with probability distribution *p*(*λ*) determines both Alice’s statistics *p*(*a*∣*X*, *λ*) and Bob’s local state ${\sigma}_{\lambda}^{{{{\rm{B}}}}}$, leading to the assemblage ${{{\mathcal{A}}}} (a,X)={\sum }_{\lambda } p(a| X, \lambda )p (\lambda ){\sigma }_{\lambda }^{{{{\rm{B}}}}}$. Inequality (1) holds for arbitrary estimators and measurement settings whenever a LHS model exists. The sharpest formulation of Eq. (1) is thus obtained by optimising these choices to minimise the estimation error. The optimal estimator ${h}_{{{{\rm{est}}}}}(a)={{{\rm{Tr}}}}[{\rho }_{a| X}^{{{{\rm{B}}}}}H]$ attains the lower bound^3^ ${{{\rm{Var}}}}[{H}_{{{{\rm{est}}}}}]\ge {\sum }_{a}p(a| X){{{\rm{Var}}}}[{\rho }_{a| X}^{{{{\rm{B}}}}},H]$, where ${{{\rm{Var}}}}[\rho ,H]={\langle {H}^{2}\rangle }_{\rho }-{\langle H\rangle }_{\rho }^{2}$ is the variance with ${\langle O\rangle }_{\rho }={{{\rm{Tr}}}}[\rho O]$. Optimising over Alice’s measurement setting *X* leads to the quantum conditional variance

2 $${{{{\rm{Var}}}}}_{{{{\rm{Q}}}}}^{{{{\rm{B}}}}| {{{\rm{A}}}}}[{{{\mathcal{A}}}},H]:=\mathop{{{\mathrm{min}}}}_{X}\, \mathop{\sum} _{a}p(a| X){{{\rm{Var}}}}[{\rho }_{a| X}^{{{{\rm{B}}}}},H],$$

and the optimised version of Reid’s condition (1) reads ${{{{\rm{Var}}}}}_{{{{\rm{Q}}}}}^{{{{\rm{B}}}}| {{{\rm{A}}}}}[{{{\mathcal{A}}}},H]{{{{\rm{Var}}}}}_{{{{\rm{Q}}}}}^{{{{\rm{B}}}}| {{{\rm{A}}}}}[{{{\mathcal{A}}}},M]\ge | {\langle [H,M]\rangle }_{{\rho }^{{{{\rm{B}}}}}}{| }^{2}/4$. The uncertainty-based detection of the EPR paradox is based on the fact that Alice’s choice of measurement can steer Bob’s system into conditional states that have small variances for either one of the two non-commuting observables *H* and *M*.

### EPR-assisted metrology

To express quantum mechanical complementarity in the framework of quantum metrology^10–13^, we assume that the observable *H* imprints a local phase shift *θ* on Bob’s system through the unitary evolution *e*^−*iHθ*^—see Fig. 1b. The phase shift *θ* is complementary to the generating observable *H* and we show that the violation of

3 $${{{\rm{Var}}}}[{\theta }_{{{{\rm{est}}}}}]{{{\rm{Var}}}}[{H}_{{{{\rm{est}}}}}]\ge \frac{1}{4n}$$

implies an EPR paradox and reveals steering from *A* to *B*. Here, Var[*θ*_est_] describes the error of an arbitrary estimator for the phase *θ*, constructed from local measurements by Alice and Bob on *n* copies of their state. Given any *M*, it is possible to construct an estimator *θ*_est_ that achieves in the central limit (*n* ≫ 1)

4 $${{{\rm{Var}}}}[{\theta }_{{{{\rm{est}}}}}]=\frac{{{{\rm{Var}}}}[{M}_{{{{\rm{est}}}}}]}{n| {\langle [H,M]\rangle }_{{\rho }^{{{{\rm{B}}}}}}{| }^{2}}.$$

Essentially, we convert an *n*-sample average of *M*_est_ into an estimate of *θ* (see “Methods” for details). For this specific estimation strategy, we thus recover the uncertainty-based formulation (1) of the EPR paradox from the more general expression (3).

In the following, we will derive our main result, which will allow us to prove the above statements. First note that the local phase shift acts on Bob’s conditional quantum states but has no impact on Alice’s measurement statistics due to no-signalling, and thus produces the assemblage ${{{{\mathcal{A}}}}}_{\theta }(a,X)=p(a| X){\rho }_{a| X,\theta }^{{{{\rm{B}}}}}$, where ${\rho }_{a| X,\theta }^{{{{\rm{B}}}}}={e}^{-iH\theta }{\rho }_{a| X}^{{{{\rm{B}}}}}{e}^{iH\theta }$. This implies the phase shift has no impact on the existence of LHS models, and ${{{{\mathcal{A}}}}}_{\theta } (a,X)= {\sum }_{\lambda } p(a| X, \lambda )p (\lambda ) {\sigma}_{\lambda ,\theta }^{{{{\rm{B}}}}}$. Without any assistance from Alice, Bob’s precision of the estimation of *θ* is determined by his reduced density matrix ${\rho }_{\theta }^{{{{\rm{B}}}}}$. In this case, the error of an arbitrary unbiased estimator ${\theta }_{{{{\rm{est}}}}}^{{{{\rm{B}}}}}$ for *θ* is bounded by the quantum Cramér–Rao bound, ${{{\rm{Var}}}}[{\theta }_{{{{\rm{est}}}}}^{{{{\rm{B}}}}}]\ge {(n{F}_{{{{\rm{Q}}}}}[{\rho }^{{{{\rm{B}}}}},H])}^{-1}$, the central theorem of quantum metrology^11–13,19–21^, where *F*_Q_[*ρ*^B^, *H*] is the QFI. *F*_Q_[*ρ*^B^, *H*] can be thought of intuitively as measuring the sensitivity of the state *ρ*^B^ to evolution generated by *H*; see “Methods” for a formal definition and explicit expression in terms of the eigenvectors and eigenvalues of *ρ*^B^. The quantum Cramér-Rao bound can be saturated by optimising both the estimator and the measurement observable^21^.

In the assisted phase-estimation protocol, Fig. 1b, Alice communicates to Bob her measurement setting and result, *i.e.* *X* and *a*. This additional knowledge allows Bob to adapt the choice of his observable as a function of the conditional state ${\rho }_{a| X}^{{{{\rm{B}}}}}$ and to achieve the maximal sensitivity ${F}_{{{{\rm{Q}}}}}[{\rho }_{a| X}^{{{{\rm{B}}}}},H]$ for an estimation of *θ*. This way, he can attain an average sensitivity as large as the quantum conditional Fisher information

5 $${F}_{{{{\rm{Q}}}}}^{{{{\rm{B}}}}| {{{\rm{A}}}}}[{{{\mathcal{A}}}},H]:=\mathop{{{\mathrm{max}}}}_{X}\, \mathop{\sum}_{a}p(a| X){F}_{{{{\rm{Q}}}}}[{\rho }_{a| X}^{{{{\rm{B}}}}},H].$$

As the main result of our paper, we show that in the absence of steering the quantum conditional Fisher information (5) is always bounded from above in terms of the quantum conditional variance (2): For any assemblage ${{{\mathcal{A}}}}$ that admits a LHS model, the following bound holds:

6 $${F}_{{{{\rm{Q}}}}}^{{{{\rm{B}}}}| {{{\rm{A}}}}}[{{{\mathcal{A}}}},H]\le 4{{{{\rm{Var}}}}}_{{{{\rm{Q}}}}}^{{{{\rm{B}}}}| {{{\rm{A}}}}}[{{{\mathcal{A}}}},H].$$

The proof (see “Methods”) primarily follows from the fact that the QFI *F*_Q_[*ρ*, *H*] is a convex function of the state *ρ*, while the variance Var[*ρ*, *H*] is instead concave^22^. Note that *F*_Q_[*ρ*^B^, *H*] ≤ 4Var[*ρ*^B^, *H*] holds for arbitrary *ρ*^B^, and by means of the Cramér–Rao bound implies the phase-generator complementarity relation

7 $${{{\rm{Var}}}}[{\theta }_{{{{\rm{est}}}}}^{{{{\rm{B}}}}}]{{{\rm{Var}}}}[{\rho }^{{{{\rm{B}}}}},H]\ge \frac{1}{4n}.$$

This clearly shows how a violation of (3) implies an EPR paradox. The result (6) has several important consequences that we discuss in the remainder of this article.

Useful steering for quantum metrology is identified by correlations that violate the condition (6). We note that classical correlations between Alice and Bob may be sufficient for having ${F}_{{{{\rm{Q}}}}}[{\rho }^{{{{\rm{B}}}}},H]\, < \,{F}_{{{{\rm{Q}}}}}^{{{{\rm{B}}}}| {{{\rm{A}}}}}[{\rho }^{{{{\rm{AB}}}}},H]$ and ${{{{\rm{Var}}}}}_{{{{\rm{Q}}}}}^{{{{\rm{B}}}}| {{{\rm{A}}}}}[{\rho }^{{{{\rm{AB}}}}},H]\, < \,{{{\rm{Var}}}}[{\rho }^{{{{\rm{B}}}}},H]$. This shows that assistance is useful even in the absence of steering to improve the estimation precision for *θ* and *H*, but only with steering can the limit defined by quantum mechanical complementarity (6) be overcome.

### Comparison to Reid-type criteria

The metrological steering condition (6) is stronger than standard criteria based on Heisenberg–Robertson uncertainty relations. In fact, the lower bound

8 $$\frac{| {\langle [H,M]\rangle }_{{\rho }^{{{{\rm{B}}}}}}{| }^{2}}{{{{{\rm{Var}}}}}_{{{{\rm{Q}}}}}^{{{{\rm{B}}}}| {{{\rm{A}}}}}[{{{\mathcal{A}}}},M]}\le {F}_{{{{\rm{Q}}}}}^{{{{\rm{B}}}}| {{{\rm{A}}}}}[{{{\mathcal{A}}}},H]$$

holds for arbitrary observables *H*, *M* and, besides no-signalling, does not require assumptions about the assemblage ${{{\mathcal{A}}}}$ (see “Methods” for the proof). Hence, the bound (6) implies Reid’s uncertainty-based condition (1) for all LHS models. In experimentally relevant situations where the observables *H* and *M* are chosen as linear observables, such as quadrature measurements in quantum optics or collective spins in atomic systems, the bound (8) can be interpreted as a Gaussian approximation to the assisted sensitivity. In fact, violation of criterion (1) (choosing the appropriate observables) is necessary and sufficient for steering of Gaussian states by Gaussian measurements^4^. The criterion (1) is also able to detect the steering of some non-Gaussian states^23,24^, but its ability to capture complex distributions is ultimately limited by only considering first and second moments. The metrological approach thus provides particular advantages for the highly challenging problem of steering detection in non-Gaussian quantum states. This is in analogy to the metrological detection of entanglement that is known to be significantly more efficient in terms of the QFI instead of Gaussian quantifiers such as spin squeezing coefficients^13,16,25,26^.

### Bounds for specific measurements

Experimental tests of the condition (6) are possible even without knowledge of the measurement settings that achieve the optimisations in Eqs. (5) and (2). Any fixed choice of local measurement settings *X* and $X^{\prime}$ for Alice and Bob, respectively, provides a joint sensitivity quantified by the (classical) Fisher information ${F}^{{{{\rm{AB}}}}}[{{{{\mathcal{A}}}}}_{\theta },X,X^{\prime} ]$, and we obtain the hierarchy of inequalities (see “Methods” for a proof)

9 $$\frac{1}{n{{{\rm{Var}}}}[{\theta }_{{{{\rm{est}}}}}]}\le {F}^{{{{\rm{AB}}}}}[{{{{\mathcal{A}}}}}_{\theta },X,X^{\prime} ]\le {F}_{{{{\rm{Q}}}}}^{{{{\rm{A}}}},{{{\rm{B}}}}}[{{{{\mathcal{A}}}}}_{\theta }]\le {F}_{{{{\rm{Q}}}}}^{{{{\rm{B}}}}| {{{\rm{A}}}}}[{{{\mathcal{A}}}},H],$$

where ${F}_{{{{\rm{Q}}}}}^{{{{\rm{A}}}},{{{\rm{B}}}}}[{{{{\mathcal{A}}}}}_{\theta }]={\max }_{X,X^{\prime} }{F}^{{{{\rm{AB}}}}}[{{{{\mathcal{A}}}}}_{\theta },X,X^{\prime} ]$ is the joint Fisher information, maximised over local measurement settings. Similarly, any fixed choice of *X* yields an upper bound on (2) and the inequalities

10 $${{{{\rm{Var}}}}}_{{{{\rm{Q}}}}}^{{{{\rm{B}}}}| {{{\rm{A}}}}}[{{{\mathcal{A}}}},H]\le \mathop{\sum} _{a}p(a| X){{{\rm{Var}}}}[{\rho }_{a| X}^{{{{\rm{B}}}}},H]\le {{{\rm{Var}}}}[{H}_{{{{\rm{est}}}}}]$$

are saturated by an optimal measurement (2) and estimator, respectively^3^. These hierarchies reveal that any choice of local measurement settings leads to experimentally observable bounds for both sides of the inequality (6). They further show how the simpler condition (3) can be derived from (6). Note that a different choice of setting *X* must be used for estimating *θ* or *H* in order to observe any effect from steering correlations. Both parties generally need to know which of the two settings is being used.

### Bounds on ${F}_{{{{\rm{Q}}}}}^{{{{\rm{B}}}}| {{{\rm{A}}}}}$ and ${{{{\rm{Var}}}}}_{{{{\rm{Q}}}}}^{{{{\rm{B}}}}| {{{\rm{A}}}}}$

It is interesting to note that both sides of the inequality (6) respect the same upper and lower bounds

11 $$	{F}_{{{{\rm{Q}}}}}[{\rho}^{{{\rm{B}}}}, H] \quad \,\le \quad \,{F}_{{{\rm{Q}}}}^{{{\rm{B}}}| {{{\rm{A}}}}}[{{{\mathcal{A}}}}, H] \qquad \mathop{\le}\limits^{(*)} \quad 4{{{\rm{Var}}}}[{\rho}^{{{\rm{B}}}}, H],\\ 	{F}_{{{\rm{Q}}}}[{\rho }^{{{\rm{B}}}}, H] \quad \mathop{\le}\limits^{(*)} \quad 4 {{{{\rm{Var}}}}}_{{{{\rm{Q}}}}}^{{{{{\rm{B}}}}| {{{\rm{A}}}}}}[{{{\mathcal{A}}}}, H]\quad\,\le\, \quad 4{{{\rm{Var}}}} [{\rho}^{{{\rm{B}}}},H].$$

These inequalities hold for arbitrary assemblages ${{{\mathcal{A}}}}$.

When we can assume Alice’s system to be quantum, we obtain the assemblage ${{{\mathcal{A}}}}$ from the bipartite quantum state *ρ*^AB^ as ${{{\mathcal{A}}}}(a,X)={{{{\rm{Tr}}}}}_{{{{\rm{A}}}}}[{E}_{a| X}^{{{{\rm{A}}}}}{\rho }^{{{{\rm{AB}}}}}]$, where the ${E}_{a| X}^{{{{\rm{A}}}}}\ge 0$ form a positive operator-valued measure (POVM) for the measurement setting *X*, normalised by ${\sum }_{a}{E}_{a| X}^{{{{\rm{A}}}}}={{\mathbb{1}}}^{{{{\rm{A}}}}}$. The inequalities in (11) marked by (*) are saturated when *ρ*^AB^ is a pure state, assuming Alice is able to perform any quantum measurement (see “Methods”). This result is a consequence of the remarkable facts that the QFI is the convex roof of the variance^27^ while the variance is its own concave roof^22^, in addition to Alice being able to steer Bob’s system into any pure-state ensemble for the local state *ρ*^B^^28^.

We construct explicit measurement bases for Alice to achieve steering in the optimal ensembles that saturate the above inequalities (Supplementary Note 5). We further observe that the inequality (6), even with a fixed generator *H*, is capable of witnessing steering correlations for almost any pure state *ψ*^AB^. More precisely, (6) is violated for any entangled *ψ*^AB^ whenever *H* is not constant on the support of the local state *ρ*^B^.

### Steering of GHZ states

Let us illustrate our criterion with a simple but relevant example. Consider a system composed of *N* + 1 qubits, partitioned into a single control qubit (Alice) and the remaining *N* qubits on Bob’s side, that are prepared in a Greenberger–Horne–Zeilinger (GHZ) state of the form

12 $$\left|{{{{\rm{GHZ}}}}}_{\phi }^{N+1}\right\rangle =\frac{1}{\sqrt{2}}\left(\left|0\right\rangle \otimes {\left|0\right\rangle }^{\otimes N}+{e}^{i\phi }\left|1\right\rangle \otimes {\left|1\right\rangle }^{\otimes N}\right),$$

where $\left|0\right\rangle ,\left|1\right\rangle$ are eigenstates of the Pauli matrix *σ*_*z*_. We take the local Hamiltonian ${J}_{z}^{{{{\rm{B}}}}}=\frac{1}{2}{\sum }_{i\in B}{\sigma }_{z}^{(i)}$, where the sum extends over the particles on Bob’s side. When Alice measures her qubit in the *σ*_*z*_ basis, Bob attains the quantum conditional variance ${{{{\rm{Var}}}}}_{{{{\rm{Q}}}}}^{{{{\rm{B}}}}| {{{\rm{A}}}}}[|{{{{\rm{GHZ}}}}}_{\phi }^{N+1}\rangle ,{J}_{z}^{{{{\rm{B}}}}}]=0$. GHZ states have the property^29^ $|{{{{\rm{GHZ}}}}}_{\phi }^{N+1}\rangle \!=\!\frac{1}{\sqrt{2}}(|+\rangle \otimes |{{{{\rm{GHZ}}}}}_{\phi }^{N}\rangle +|-\rangle \otimes |{{{{\rm{GHZ}}}}}_{\phi +\pi }^{N}\rangle )$, where $\left|+\right\rangle ,\left|-\right\rangle$ are eigenstates of *σ*_*x*_. This allows Alice to steer Bob’s system into GHZ states by measuring in the *σ*_*x*_ basis, and we obtain

13 $${F}_{{{{\rm{Q}}}}}^{{{{\rm{B}}}}| {{{\rm{A}}}}}\left[\left|{{{{\rm{GHZ}}}}}_{\phi }^{N+1}\right\rangle ,{J}_{z}\right]=\frac{1}{2}\left({F}_{{{{\rm{Q}}}}}\left[\left|{{{{\rm{GHZ}}}}}_{\phi }^{N}\right\rangle ,{J}_{z}^{{{{\rm{B}}}}}\right]+{F}_{{{{\rm{Q}}}}}\left[\left|{{{{\rm{GHZ}}}}}_{\phi +\pi }^{N}\right\rangle ,{J}_{z}^{{{{\rm{B}}}}}\right]\right)={N}^{2}.$$

This measurement is optimal and achieves the maximum in (5) since ${F}_{{{{\rm{Q}}}}}[\rho ,{J}_{z}^{{{{\rm{B}}}}}]\le {N}^{2}$ holds for arbitrary quantum states^12,13^. Steering is detected by the clear violation of the condition (6) for LHS models. So far the only known criteria able to detect steering in multipartite GHZ states are based on nonlocal observables that require individual addressing of the particles (see e.g. refs. ^30,31^), while our criterion is accessible by collective measurements. The criterion is moreover robust to white noise: For a mixture $\rho =p|{{{{\rm{GHZ}}}}}_{\phi }^{N+1}\rangle \langle {{{{\rm{GHZ}}}}}_{\phi }^{N+1}|+(1-p){\mathbb{1}}/{2}^{N+1}$, using the same measurements we obtain ${F}_{{{{\rm{Q}}}}}^{{{{\rm{B}}}}| {{{\rm{A}}}}}[\rho ,{J}_{z}]\ge {p}^{2}{N}^{2}/[p+2(1-p)/{2}^{N}], 4{{{{\rm{Var}}}}}_{{{{\rm{Q}}}}}^{{{{\rm{B}}}}| {{{\rm{A}}}}}[\rho ,{J}_{z}]\le (1-p)N+p(1-p){N}^{2}$. For large *N*, whenever *p* ⪆ ${1}{/} {\sqrt{N}}$, the criterion witnesses steering. See Supplementary Note 2 for details and Supplementary Note 3 for an additional example involving a Schrödinger cat state.

### Steering of atomic split twin Fock states

As an example of immediate practical relevance for state-of-the-art ultracold-atom experiments, consider *N*/2 spin excitations symmetrically distributed over *N* particles, *i.e.*, a twin Fock state. Separating the particles into two addressable modes A and B with a 50:50 beam splitter results in a split twin Fock state $\left|{{{{\rm{STF}}}}}_{N}\right\rangle$, which has been generated experimentally^32^. Similar experiments based on squeezed states were able to use Reid’s criterion to verify steering^33,34^, but the vanishing polarisation ${\langle {J}_{x}^{{{{\rm{B}}}}}\rangle }_{{\rho }^{{{{\rm{B}}}}}}={\langle {J}_{y}^{{{{\rm{B}}}}}\rangle }_{{\rho }^{{{{\rm{B}}}}}}=0$ makes this challenging for split twin Fock states and so far only the entanglement between A and B could be detected^32^. We show that the criterion (6) successfully reveals the EPR steering of split twin Fock states when Alice measures local spin observables ${J}_{x}^{{{{\rm{A}}}}}$, ${J}_{z}^{{{{\rm{A}}}}}$ and a phase shift *θ* is generated by ${J}_{z}^{{{{\rm{B}}}}}$. We obtain ${{{{\rm{Var}}}}}_{{{{\rm{Q}}}}}^{{{{\rm{B}}}}| {{{\rm{A}}}}}[|{{{{\rm{STF}}}}}_{N}\rangle ,{J}_{z}^{{{{\rm{B}}}}}]=0$ and ${F}_{{{{\rm{Q}}}}}^{{{{\rm{B}}}}| {{{\rm{A}}}}}[|{{{{\rm{STF}}}}}_{N}\rangle ,{J}_{z}^{{{{\rm{B}}}}}]=N/4$, leading to a violation of (6) that scales linearly with *N*. This value is limited by the partition noise that is introduced by the beam splitter which generates binomial fluctuations of the particle number in each mode.

**Fig. 2:**
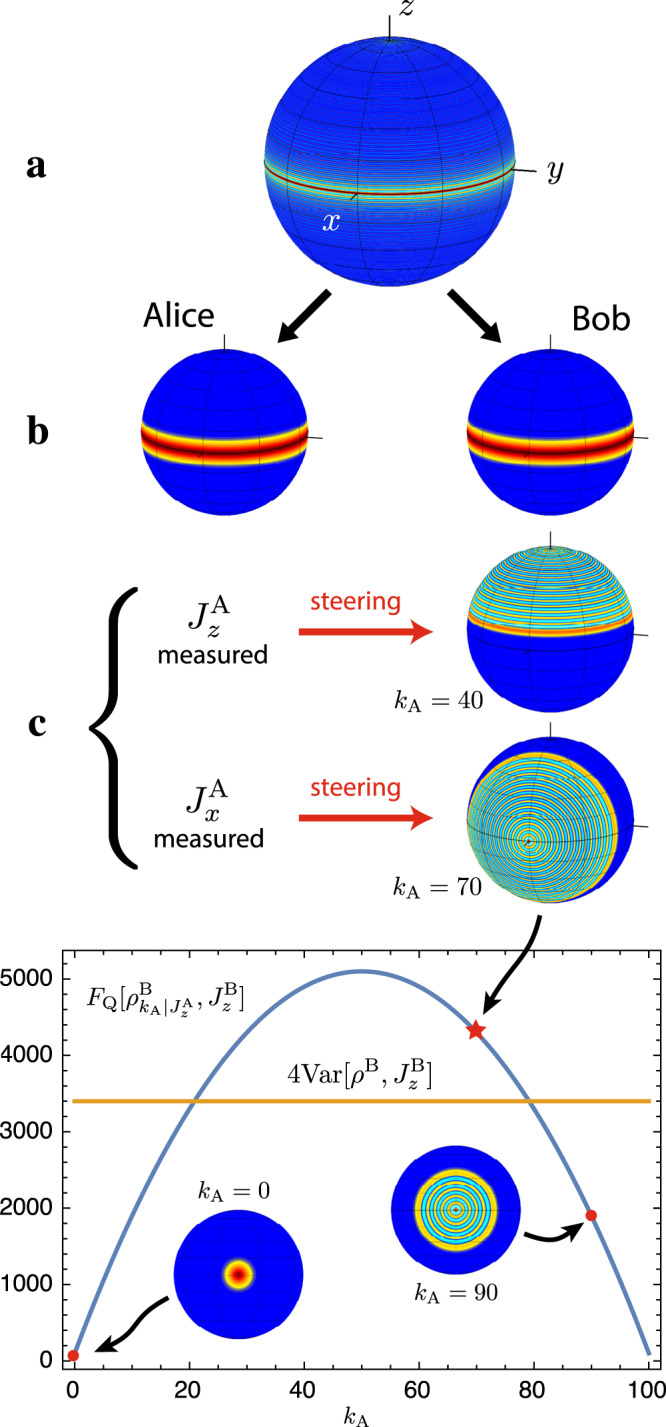
EPR-assisted metrology with twin Fock states. **a** We consider a twin Fock state with *N* = 200 particles, that is split into two parts with *N*_A_ = *N*_B_ = *N*/2, here represented by the Wigner function on the Bloch sphere. **b** The reduced state on either side is a mixture of Dicke states, resulting from tracing out the other half of the system. **c** The two subsystems show perfect correlations for both measurement settings *J*_*x*_ and *J*_*z*_: When Alice measures ${J}_{z}^{{{{\rm{A}}}}}$ (${J}_{x}^{{{{\rm{A}}}}}$) and obtains the result *k*_A_, she steers Bob’s system into an eigenstate of ${J}_{z}^{{{{\rm{B}}}}}$ (${J}_{x}^{{{{\rm{B}}}}}$) with eigenvalue *N*/2 − *k*_A_. This can be used for assisted quantum metrology, and to reveal an EPR paradox. In the plot we show Bob’s sensitivity ${F}_{{{{\rm{Q}}}}}[{\rho }_{{k}_{{{{\rm{A}}}}}| {J}_{x}^{{{{\rm{A}}}}}}^{{{{\rm{B}}}}},{J}_{z}^{{{{\rm{B}}}}}]$ when Alice obtains the result *k*_A_ from measuring ${J}_{x}^{{{{\rm{A}}}}}$ (blue line). Alice’s results are all equally probable with $p({k}_{{{{\rm{A}}}}}| {J}_{x}^{{{{\rm{A}}}}})=2/(N+2)$. Bob’s average sensitivity ${F}_{{{{\rm{Q}}}}}^{{{{\rm{B}}}}| {{{\rm{A}}}}}[|{{{{\rm{SD}}}}}_{N,N/2}\rangle ,{J}_{z}^{{{{\rm{B}}}}}]$ coincides with the variance for the reduced state $4{{{\rm{Var}}}}[{\rho }^{{{{\rm{B}}}}},{J}_{z}^{{{{\rm{B}}}}}]$ (yellow line), indicating that the measurement is optimal (Supplementary Note 4).

To overcome this limit, we propose the following alternative preparation of split Dicke states. Consider two addressable groups of *N*/2 atoms each. A collective measurement of the total number *k* of spin excitations projects the system into a split Dicke state $|{{{{\rm{SD}}}}}_{N,k}\rangle$ without partition noise. This can be realised, e.g., with arrays of cold atoms in a cavity^35^. Using the same settings for Alice and Bob as before, these states still yield ${{{{\rm{Var}}}}}_{{{{\rm{Q}}}}}^{{{{\rm{B}}}}| {{{\rm{A}}}}}[|{{{{\rm{SD}}}}}_{N,k}\rangle ,{J}_{z}^{{{{\rm{B}}}}}]=0$ while leading to significantly larger values of the quantum conditional Fisher information, and for the twin Fock case, *k* = *N*/2, we obtain the quadratic scaling ${F}_{{{{\rm{Q}}}}}^{{{{\rm{B}}}}| {{{\rm{A}}}}}[|{{{{\rm{SD}}}}}_{N,N/2}\rangle ,{J}_{z}^{{{{\rm{B}}}}}]=N(N+4)/12$; see Fig. 2. For details on arbitrary split Dicke states with and without partition noise, see Supplementary Note 4.

### Phase estimation with multiple generators

Our result reveals the role of steering for generating probe states that are highly sensitive to the evolutions generated by a family of non-commuting generators, **H** = (*H*_1_, …, *H*_*m*_). We focus on a sequential scenario, where in each experimental trial a single parameter is generated by one of the elements of **H**. Bob’s estimation of the phase is assisted by steering from Alice who picks different measurement settings *X*_*i*_ as a function of the acting Hamiltonian *H*_*i*_ and includes details of *H*_*i*_ in her communication to Bob. Achieving high sensitivity for multiple generators is relevant for multiparameter quantum metrology^36–41^, but the identification of a single measurement observable that is suitable for all parameters^42,43^ provides an additional complication that is not considered in our scenario.

A suitable figure of merit for Bob’s average sensitivity is

14 $$\overline{{F}_{{{{\rm{Q}}}}}^{{{{\rm{B}}}}| {{{\rm{A}}}}}[{{{\mathcal{A}}}},{{{\bf{H}}}}]}=\mathop{\sum }\limits_{i=1}^{m}{F}_{{{{\rm{Q}}}}}^{{{{\rm{B}}}}| {{{\rm{A}}}}}[{{{\mathcal{A}}}},{H}_{i}].$$

Using the same techniques as for the main inequality (6), we find that any assemblage admitting a LHS model satisfies (see “Methods”)

15 $$\overline{{F}_{{{{\rm{Q}}}}}^{{{{\rm{B}}}}| {{{\rm{A}}}}}[{{{\mathcal{A}}}},{{{\bf{H}}}}]}\le \mathop{\max}\limits_{{\left|\phi \right\rangle }^{{{{\rm{B}}}}}}\mathop{\sum }\limits_{i=1}^{m}4{{{\rm{Var}}}}[{\left|\phi \right\rangle^{{{{\rm{B}}}}}},{H}_{i}].$$

An advantage over (6) is that the right-hand side is state-independent. For a system *B* of dimension *d*, we can take the *H*_*i*_ to be a set of *d*^2^ − 1 Hilbert–Schmidt orthonormal generators of SU(*d*), and this bound simplifies to

16 $$\overline{{F}_{{{{\rm{Q}}}}}^{{{{\rm{B}}}}| {{{\rm{A}}}}}[{{{\mathcal{A}}}},{{{\bf{H}}}}]}\le 4(d-1).$$

As a simple example, when Bob has a qubit (*d* = 2), we can take the Pauli matrices as generators, ${H}_{i}={\sigma }_{i}/\sqrt{2},\ i=x,y,z$. Then (16) becomes $\overline{{F}_{{{{\rm{Q}}}}}^{{{{\rm{B}}}}| {{{\rm{A}}}}}[{{{\mathcal{A}}}},{{{\bf{H}}}}]}\le 4$. For a shared maximally entangled state, this inequality is violated since $\overline{{F}_{{{{\rm{Q}}}}}^{{{{\rm{B}}}}| {{{\rm{A}}}}}[{{{\mathcal{A}}}},{{{\bf{H}}}}]}=6$. To interpret these numbers, note that any pure qubit state on Bob’s side is optimal for sensing rotations about two orthogonal axes (each of which contributes a QFI of 2), but useless for the remaining axis. With a maximally entangled state, Alice can choose to steer Bob’s system into a state that is optimal for whichever axis has been chosen, thus sensing about any given axis is optimal.

### Steering quantification

We may expect that the degree of violation of (6), in a suitable sense, measures the amount of steering correlations. The proposed resource theory of steering^7^ gives a set of criteria to be satisfied by a valid measure of steering. A general *steering monotone* ${{{\mathcal{S}}}}$ assigns a non-negative real number to each assemblage. First, we require (i) ${{{\mathcal{S}}}}({{{\mathcal{A}}}})=0$ for every assemblage ${{{\mathcal{A}}}}$ with a LHS model. Next, (ii) ${{{\mathcal{S}}}}$ must be non-increasing (on average) when ${{{\mathcal{A}}}}$ is operated upon by local operations and one-way classical communication from Bob to Alice (1W-LOCC). Finally, we may optionally require (iii) convexity: ${{{\mathcal{S}}}}(p{{{{\mathcal{A}}}}}_{1}+[1-p]{{{{\mathcal{A}}}}}_{2})\le p{{{\mathcal{S}}}}({{{{\mathcal{A}}}}}_{1})+(1-p){{{\mathcal{S}}}}({{{{\mathcal{A}}}}}_{2})$ for any pair of assemblages classically mixed with probability *p*. Here, we propose two potential quantifiers and address whether they satisfy these criteria.

One quantity is the maximum possible violation of (6), given the ability to vary the generator *H*. Since a rescaling of *H* → *rH* scales the QFI and the variance by the same factor *r*^2^, we fix the norm of *H* – a convenient choice is to take ${{{\rm{Tr}}}}[{H}^{2}]=1$. Then the maximum violation of (6) is

17 $${{{\mathcal{S}}}}_{{{\mathrm{max}}}}({{{\mathcal{A}}}}):=\mathop{{{\mathrm{max}}}}_{H, {{{\rm{Tr}}}}[{H}^{2}]=1}{\left[\frac{1}{4}{F}_{{{{\rm{Q}}}}}^{{{{\rm{B}}}}| {{{\rm{A}}}}}[{{{\mathcal{A}}}},H]-{{{{\rm{Var}}}}}_{{{{\rm{Q}}}}}^{{{{\rm{B}}}}| {{{\rm{A}}}}}[{{{\mathcal{A}}}},H]\right]}^{+},$$

where ${\left[x\right]}^{+}=\max \{0,x\}$. For a bipartite pure quantum state *ψ*^AB^, we have the easily computable formula (Supplementary Note 6) ${{{{\mathcal{S}}}}}_{\max }({\psi }^{{{{\rm{AB}}}}})={\lambda }_{\max }[{{{\rm{diag}}}}({{{\bf{p}}}})-{{{\bf{p}}}}{{{{\bf{p}}}}}^{T}]$, where **p** is the vector of eigenvalues of *ρ*^B^ (equivalently, the Schmidt coefficients of *ψ*^AB^) and ${\lambda }_{\max }$ denotes the largest eigenvalue.

Alternatively, we can average over all *H* with ${{{\rm{Tr}}}}[{H}^{2}]=1$. Formally, this (rescaled) average is defined by

18 $${{{{\mathcal{S}}}}}_{{{{\rm{avg}}}}}({{{\mathcal{A}}}})=({d}^{2}-1){\left[\int\mu ({{{\rm{d}}}}{{{\bf{n}}}})\frac{1}{4}{F}_{{{{\rm{Q}}}}}^{{{{\rm{B}}}}| {{{\rm{A}}}}}[{{{\mathcal{A}}}},{{{\bf{n}}}}\cdot {{{\bf{H}}}}]-{{{{\rm{Var}}}}}_{{{{\rm{Q}}}}}^{{{{\rm{B}}}}| {{{\rm{A}}}}}[{{{\mathcal{A}}}},{{{\bf{n}}}}\cdot {{{\bf{H}}}}]\right]}^{+},$$

where *H*_*i*_ is any basis of orthonormal SU(*d*) generators, and *μ* is the uniform measure over the sphere of unit vectors ∣**n**∣ = 1. For pure states, we have ${{{{\mathcal{S}}}}}_{{{{\rm{avg}}}}}({\psi }^{{{{\rm{AB}}}}})={\sum }_{i\ne j}{p}_{i}{p}_{j}(1+\frac{2}{{p}_{i}+{p}_{j}})$.

It follows immediately from (6) that both ${{{{\mathcal{S}}}}}_{\max }$ and ${{{{\mathcal{S}}}}}_{{{{\rm{avg}}}}}$ satisfy criterion (i). Moreover, we find that both are faithful indicators for pure states, meaning that they each vanish if and only if *ψ*^AB^ is separable. Convexity is also straightforward to prove (see Supplementary Note 6 for all details). Criterion (ii) can be ruled out for ${{{{\mathcal{S}}}}}_{\max }$ by again considering pure states: in this case, steering correlations (as with all correlations) are equivalent to entanglement^4,44^. ${{{{\mathcal{S}}}}}_{\max }$ is found not to be an entanglement monotone; nevertheless, it remains an important quantity to consider if one is interested in observing the maximum possible violation. On the other hand, we prove that ${{{{\mathcal{S}}}}}_{{{{\rm{avg}}}}}$ is a pure state entanglement monotone. Thus it remains an open question whether ${{{{\mathcal{S}}}}}_{{{{\rm{avg}}}}}$ is in general a steering monotone.

## Discussion

We formulated the EPR paradox in the framework of quantum metrology, showing that it can be interpreted as an apparent violation of the complementarity relation between a local phase shift and its generator. This idea allowed us to derive a criterion to detect EPR correlations which is based on the QFI, and thus stronger than known criteria based on the Heisenberg uncertainty relation. We illustrated this with concrete examples of non-Gaussian states in optical and atomic systems that are of immediate interest for experimental studies. By expressing the EPR paradox as a metrological task, our results demonstrate that such correlations can be useful for quantum-enhanced measurement protocols, thus having the potential to enable new sensing applications in quantum technologies.

## Methods

### Fisher information

For a probability distribution *p*(*x*∣*θ*) parameterised by $\theta \in {\mathbb{R}}$, the classical Fisher information is $F[p(x| \theta )]:=\int {{{\rm{d}}}}x\ p(x| \theta ){\left[{\partial }_{\theta }{{{\mathrm{ln}}}}\,p(x| \theta )\right]}^{2}$. The quantum version *F*_Q_[*ρ*_*θ*_] for a parameter-dependent state *ρ*_*θ*_ may be defined as the maximum classical Fisher information associated with statistics obtained from any possible POVM {*E*_*x*_} via $p(x| \theta )={{{\rm{Tr}}}}[{\rho }_{\theta }{E}_{x}]$^21^. In the case of unitary parameter encoding *ρ*_*θ*_ = *e*^−*iθH*^*ρe*^*iθH*^ with a fixed generator *H*, the QFI is independent of *θ*, so we denote it by *F*_Q_[*ρ*, *H*]. This can be computed from the eigenvectors $\left|{\psi }_{i}\right\rangle$ and eigenvalues *λ*_*i*_ of *ρ*:

19 $${F}_{{{{\rm{Q}}}}}[\rho ,H]=2\mathop{\sum} _{i,j:\ {\lambda }_{i}+{\lambda }_{j}\ne 0}\frac{{({\lambda }_{i}-{\lambda }_{j})}^{2}}{{\lambda }_{i}+{\lambda }_{j}}\left| \langle {\psi }_{i}|H|{\psi }_{j}\rangle \right| ^{2}.$$

### Proof of the main result

Suppose ${{{\mathcal{A}}}}$ is described by a LHS model, then

20 $${F}_{{{{\rm{Q}}}}}^{{{{\rm{B}}}}| {{{\rm{A}}}}}[{{{\mathcal{A}}}},H] 	=\mathop{{{\mathrm{max}}}}_{X}\, \mathop{\sum}\limits _{a} p(a| X){F}_{{{{\rm{Q}}}}}\left[\mathop{\sum}\limits_{\lambda }\frac{p(a| X,\lambda )p(\lambda )}{p(a| X)}{\sigma }_{\lambda }^{{{{\rm{B}}}}},H\right]\\ 	\le \mathop{{{\mathrm{max}}}}_{X}\, \mathop{\sum}\limits _{a} \mathop{\sum}\limits _{\lambda } p(a| X,\lambda )p(\lambda ){F}_{{{{\rm{Q}}}}}[{\sigma }_{\lambda }^{{{{\rm{B}}}}},H]\\ 	=\mathop{\sum}\limits_{\lambda }p(\lambda ){F}_{{{{\rm{Q}}}}}[{\sigma }_{\lambda }^{{{{\rm{B}}}}},H],$$

where we used the convexity of the QFI and ∑_*a*_*p*(*a*∣*X*, *λ*) = 1, since *λ* and *X* are independent. Making use of the upper bound^13,21^ *F*_Q_[*ρ*, *H*] ≤ 4Var[*ρ*, *H*] that holds for arbitrary states *ρ*, we obtain

21 $${F}_{{{{\rm{Q}}}}}^{{{{\rm{B}}}}| {{{\rm{A}}}}}[{{{\mathcal{A}}}}, H] \le 4{\mathop{\sum}_{\lambda }}p(\lambda ){{{\rm{Var}}}}[{\sigma }_{\lambda }^{{{{\rm{B}}}}},H].$$

Moreover, following analogous steps, we obtain from the concavity of the variance^3^

22 $${{{{\rm{Var}}}}}_{{{{\rm{Q}}}}}^{{{{\rm{B}}}}| {{{\rm{A}}}}}[{{{\mathcal{A}}}},H]\ge {\mathop{\sum} _{\lambda }} p(\lambda ){{{\rm{Var}}}}[{\sigma }_{\lambda }^{{{{\rm{B}}}}},H].$$

Inserting (22) into (21) proves the result (6).

### Recovering Reid’s criterion

The QFI describes the sensitivity for a parameter *θ* generated by *H* that is achievable with an optimal measurement and estimation strategy. By using a specific estimator, constructed from the expectation value of some observable *M*, one obtains the lower bound^13,25^

23 $${F}_{{{{\rm{Q}}}}}[\rho ,H]\ge \frac{| {\langle [H,M]\rangle }_{\rho }{| }^{2}}{{{{\rm{Var}}}}[\rho ,M]}.$$

Together with the Cauchy-Schwarz inequality, we obtain for all ${{{\mathcal{A}}}}$

24 $${F}_{{{{\rm{Q}}}}}^{{{{\rm{B}}}}| {{{\rm{A}}}}}[{{{\mathcal{A}}}},H]	 \ge \mathop{{{\mathrm{max}}}}_{X}\, \mathop{\sum} _{a}p(a| X)\frac{| {\langle [H,M]\rangle }_{{\rho }_{a| X}^{{{{\rm{B}}}}}}{| }^{2}}{{{{\rm{Var}}}}[{\rho }_{a| X}^{{{{\rm{B}}}}},M]}\\ 	\ge \mathop{{{\mathrm{max}}}}_{X} \frac{| {\sum }_{a}p(a| X){\langle [H,M]\rangle }_{{\rho }_{a| X}^{{{{\rm{B}}}}}}{| }^{2}}{{\sum }_{a}p(a| X){{{\rm{Var}}}}[{\rho }_{a| X}^{{{{\rm{B}}}}},M]}\\ 	=\mathop{{{\mathrm{max}}}}_{X}\frac{| {\langle [H,M]\rangle }_{{\rho }^{{{{\rm{B}}}}}}{| }^{2}}{\mathop{\sum}\limits_{a}p(a| X){{{\rm{Var}}}}[{\rho }_{a| X}^{{{{\rm{B}}}}},M]}\\ 	=\frac{| {\langle [H,M]\rangle }_{{\rho }^{{{{\rm{B}}}}}}{| }^{2}}{{{{{\rm{Var}}}}}_{{{{\rm{Q}}}}}^{{{{\rm{B}}}}| {{{\rm{A}}}}}[{{{\mathcal{A}}}},M]}.$$

Inserting (24) into (6) yields Reid’s criterion. The formulation (1) follows by using that ${{{\rm{Var}}}}[{M}_{{{{\rm{est}}}}}]\ge {{{{\rm{Var}}}}}_{{{{\rm{Q}}}}}^{{{{\rm{B}}}}| {{{\rm{A}}}}}[{{{\mathcal{A}}}},M]$ for all *M*.

In the case of a Gaussian quantum bipartite state with Gaussian measurements by Alice, Eq. (24) can be saturated. First, note that the quadrature variances (in fact, the whole covariance matrix) are identical for each conditional state ${\rho }_{a| X}^{{{{\rm{B}}}}}$^45^. A suitable pair of conjugate quadratures *H*, *M* can be chosen such that Eq. (23) is saturated^26^, thus the first inequality in (24) is saturated. For the second inequality, note that all variances in the denominator are identical.

We can also directly recover Reid’s criterion from the weaker condition (3) by constructing a specific estimator from the measurement data *b*, *m* of Alice and Bob, respectively. We assume that the dependence of the average value ${\langle {M}_{{{{\rm{est}}}}}-M\rangle }_{\theta }={\sum }_{b}{\sum }_{m}p(b,m| Y,M,\theta )({m}_{{{{\rm{est}}}}}(b)-m)$ on *θ* is known from calibration, where $p(b,m| Y,M,\theta )=p(b| Y)\left\langle m\right|{\rho }_{b| Y,\theta }^{{{{\rm{B}}}}}\left|m\right\rangle$. Given a sample of *n* measurement results, the value of *θ* can now be estimated as the one that yields ${\langle {M}_{{{{\rm{est}}}}}-M\rangle }_{\theta }=\frac{1}{n}\mathop{\sum }\nolimits_{i = 1}^{n}({m}_{{{{\rm{est}}}}}({b}_{i})-{m}_{i})$. Without the loss of generality we calibrate the estimator around the fixed value *θ* = 0, such that the estimator for *m* is unbiased, i.e., 〈*M*_est_〉 = 〈*M*〉_*θ*=0_ (any biased estimator would lead to a larger error). The sample average evaluated at *θ* = 0 has a variance of $\frac{1}{n}{{{\rm{Var}}}}[{M}_{{{{\rm{est}}}}}]$. Note that only the distribution of Bob’s results *m*_*i*_ depends on *θ*, and therefore $| \frac{\partial }{\partial \theta }{\langle {M}_{{{{\rm{est}}}}}-M\rangle }_{\theta }| =| \frac{\partial }{\partial \theta }{\langle M\rangle }_{\theta }|$. In the central limit (*n* ≫ 1), this strategy therefore yields a sensitivity of

25 $${{{\rm{Var}}}}[{\theta }_{{{{\rm{est}}}}}]=\frac{{{{\rm{Var}}}}[{M}_{{{{\rm{est}}}}}]}{n{\left|\frac{\partial {\langle M\rangle }_{\theta }}{\partial \theta }\right|}^{2}},$$

which can be shown from a maximal likelihood analysis of the sample average distribution or from Gaussian error propagation^46^. We obtain the result Eq. (4).

### Sensitivity for fixed local measurements

For fixed measurement settings *X* and $X^{\prime}$, respectively, the joint statistics of Alice and Bob are described by the probability distribution $p(a,b| X,X^{\prime} ,\theta )=p(a| X){{{\rm{Tr}}}}[{E}_{b| X^{\prime} }{\rho }_{a| X,\theta }^{{{{\rm{B}}}}}]$ where ${E}_{b| X^{\prime} }$ is a POVM describing the measurement $X^{\prime}$. The Cramér–Rao bound

26 $$n{{{\rm{Var}}}}[{\theta }_{{{{\rm{est}}}}}]\ge 1/{F}^{{{{\rm{AB}}}}}[{{{{\mathcal{A}}}}}_{\theta },X,X^{\prime} ]$$

identifies the precision limit for any estimator that is constructed from the local measurement results *a* and *b* and for any choice of *X* and $X^{\prime}$ in terms of the Fisher information

27 $${F}^{{{{\rm{AB}}}}}[{{{{\mathcal{A}}}}}_{\theta },X,X^{\prime} ]=\mathop{\sum} _{a,b}p(a,b| X,X^{\prime} ,\theta ){\left(\frac{\partial }{\partial \theta }{{{\mathrm{ln}}}}\,p(a,b| X,X^{\prime} ,\theta )\right)}^{2}.$$

A straightforward calculation reveals that

28 $${F}^{{{{\rm{AB}}}}}[{{{{\mathcal{A}}}}}_{\theta },X,X^{\prime} ]=\mathop{\sum} _{a}p(a| X){F}^{{{{\rm{B}}}}}[X^{\prime} | {\rho }_{a| X,\theta }^{{{{\rm{B}}}}}],$$

i.e., for fixed settings, the joint sensitivity coincides with Bob’s average conditional sensitivity ${F}^{{{{\rm{B}}}}}[X^{\prime} | {\rho }_{a| X,\theta}^{{{{\rm{B}}}}}]={\sum}_{b}{{{\rm{Tr}}}}[{E}_{b| X^{\prime} }{\rho }_{a| X,\theta}^{{{{\rm{B}}}}}]{\left(\frac{\partial }{\partial \theta }{{{\mathrm{ln}}}}\,{{{\rm{Tr}}}}[{E}_{b| X^{\prime} }{\rho }_{a| X,\theta }^{{{{\rm{B}}}}}]\right)}^{2}$ since Alice’s data is independent of *θ*. Maximising over the choice of measurement yields the hierarchy

29 $${F}^{{{\rm{AB}}}} [ {{{{\mathcal{A}}}}}_{\theta}, X, X^{\prime} ] 	\le \underbrace{\mathop{{{\mathrm{max}}}}_{X} \mathop{{{\mathrm{max}}}}_{X^{\prime}}{\sum}_{a} p (a| X) {F}^{{{{\rm{B}}}}}[X^{\prime} | {\rho}_{a| X, \theta }^{{{{\rm{B}}}}}]}_{{F}_{{{{\rm{Q}}}}}^{{{{\rm{A}}}},{{{\rm{B}}}}}[{{{{\mathcal{A}}}}}_{\theta}]}\\ 	\le {\mathop{{{\mathrm{max}}}}_{X}}\mathop{\sum}\limits_{a}p(a| X)\underbrace{\mathop{{{\mathrm{max}}}}_{X^{\prime} }{F}^{{{{\rm{B}}}}}[X^{\prime} | {\rho}_{a| X,\theta }^{{{{\rm{B}}}}}]}_{{F}_{{{{\rm{Q}}}}}[{\rho}_{a| X}^{{{{\rm{B}}}}},H]}\\ 	={F}_{{{{\rm{Q}}}}}^{{{{\rm{B}}}}| {{{\rm{A}}}}}[{{{\mathcal{A}}}},H].$$

This completes the proof for the set of inequalities (9).

### Metrological steering for bipartite quantum states

Let us first note that if Alice’s system is quantum, the optimal measurements in (2) and (5) can always be implemented by rank-1 POVMs. This follows from the convexity of the QFI and the concavity of the variance (Supplementary Note 1).

Now suppose that *ρ*^AB^ is pure. Since the optimal POVM for ${F}_{{{{\rm{Q}}}}}^{{{{\rm{B}}}}| {{{\rm{A}}}}}$ is rank-1, the corresponding conditional states ${\rho }_{a| X}^{{{{\rm{B}}}}}$ are pure. An important fact about bipartite pure states is that any pure-state ensemble on Bob’s side (consistent with the average state *ρ*^B^) may be realised by an appropriate rank-1 POVM on Alice’s side^28^. Thus the optimisation can be reduced to

30 $${F}_{{{{\rm{Q}}}}}^{{{{\rm{B}}}}| {{{\rm{A}}}}}[{\rho }^{{{{\rm{AB}}}}},H] 	=\mathop{{{\mathrm{max}}}}_{{{{\{p(a),\left|{\phi }_{a}\right\rangle \}}_{a}:}\atop {{\sum }_{a}p(a)\left|{\phi }_{a}\right\rangle \left\langle {\phi }_{a}\right|={\rho }^{{{{\rm{B}}}}}}}}\ \mathop{\sum}\limits _{a} p(a){F}_{{{{\rm{Q}}}}}[\left|{\phi }_{a}\right\rangle,H]\\ 	=\mathop{{{\mathrm{max}}}}_{{{{\{p(a),\left|{\phi }_{a}\right\rangle \}}_{a}:}\atop {{\sum }_{a}p(a)\left|{\phi }_{a}\right\rangle \left\langle {\phi }_{a}\right|={\rho }^{{{{\rm{B}}}}}}}}\ 4\mathop{\sum} \limits_{a}p(a){{{\rm{Var}}}}[\left|{\phi }_{a}\right\rangle,H]\\ 	 =4{{{\rm{Var}}}}[{\rho }^{{{{\rm{B}}}}},H].$$

In the last line we used that the variance is its own concave roof^22^. For ${{{{\rm{Var}}}}}_{{{{\rm{Q}}}}}^{{{{\rm{B}}}}| {{{\rm{A}}}}}$ the minimisation is the same as taking the convex roof, resulting in^27 ^*F*_Q_[*ρ*^B^, *H*]. Hence, for a pure state *ρ*^AB^, we obtain the equalities ${F}_{{{{\rm{Q}}}}}^{{{{\rm{B}}}}| {{{\rm{A}}}}}[{\rho }^{{{{\rm{AB}}}}},H]=4{{{\rm{Var}}}}[{\rho }^{{{{\rm{B}}}}},H]$ and $4{{{{\rm{Var}}}}}_{{{{\rm{Q}}}}}^{{{{\rm{B}}}}| {{{\rm{A}}}}}[{\rho }^{{{{\rm{AB}}}}},H]={F}_{{{{\rm{Q}}}}}[{\rho }^{{{{\rm{B}}}}},H]$. For arbitrary assemblages, we obtain the upper bounds ${F}_{{{{\rm{Q}}}}}^{{{{\rm{B}}}}| {{{\rm{A}}}}}[{{{\mathcal{A}}}},H]\le 4{{{\rm{Var}}}}[{\rho }^{{{{\rm{B}}}}},H]$ and $4{{{{\rm{Var}}}}}_{{{{\rm{Q}}}}}^{{{{\rm{B}}}}| {{{\rm{A}}}}}[{{{\mathcal{A}}}},H]\ge {F}_{{{{\rm{Q}}}}}[{\rho }^{{{{\rm{B}}}}},H]$ as a consequence of convexity of the QFI, concavity of the variance, and *F*_Q_[*ρ*, *H*] ≤ 4Var[*ρ*, *H*]. For the same reason, we obtain that ${F}_{{{{\rm{Q}}}}}^{{{{\rm{B}}}}| {{{\rm{A}}}}}[{{{\mathcal{A}}}},H]\ge {F}_{{{{\rm{Q}}}}}[{\rho }^{{{{\rm{B}}}}},H]$ and ${{{{\rm{Var}}}}}_{{{{\rm{Q}}}}}^{{{{\rm{B}}}}| {{{\rm{A}}}}}[{{{\mathcal{A}}}},H]\le {{{\rm{Var}}}}[{\rho }^{{{{\rm{B}}}}},H]$ for arbitrary assemblages ${{{\mathcal{A}}}}$, including those obtained from *ρ*^AB^. This concludes the proof of (11).

### Multiple generators

One can ask whether there is a (potentially weaker) steering witness involving only the QFI. It is clear that the right-hand side of (6) cannot be made state-independent: the best one can do is to replace ${{{{\rm{Var}}}}}_{{{{\rm{Q}}}}}^{{{{\rm{B}}}}| {{{\rm{A}}}}}[{{{\mathcal{A}}}},H]$ by ${{{\mathrm{max}}}}_{\sigma }{{{\rm{Var}}}}[\sigma ,H]$, leading to an inequality that holds for all cases, even steerable.

Instead, we turn to the quantity (14). Without any assistance from Alice, the best achievable precision would be

31 $$\overline{{F}_{{{{\rm{Q}}}}}[{\rho }^{{{{\rm{B}}}}},{{{\bf{H}}}}]}:=\mathop{\sum }\limits_{i=1}^{m}{F}_{{{{\rm{Q}}}}}[{\rho }^{{{{\rm{B}}}}},{H}_{i}].$$

Following the same technique as for a single parameter, any LHS model satisfies

32 $$\overline{{F}_{{{{\rm{Q}}}}}^{{{{\rm{B}}}}| {{{\rm{A}}}}}[{{{\mathcal{A}}}},{{{\bf{H}}}}]} 	\le \overline{{F}_{{{{\rm{Q}}}}}^{* }[{{{\bf{H}}}}]}\\ 	:=\mathop{{{\mathrm{max}}}}_{{\sigma }^{{{{\rm{B}}}}}}\ \overline{{F}_{{{{\rm{Q}}}}}[{\sigma }^{{{{\rm{B}}}}},{{{\bf{H}}}}]}\\ 	=\mathop{{{\mathrm{max}}}}_{{\left|\phi \right\rangle }^{{{{\rm{B}}}}}}\ \overline{{F}_{{{{\rm{Q}}}}}[{\left|\phi \right\rangle}^{{{{\rm{B}}}}},{{{\bf{H}}}}]}\\ 	=\mathop{{{\mathrm{max}}}}_{{\left|\phi \right\rangle }^{{{{\rm{B}}}}}}\ \mathop{\sum }_{i}4{{{\rm{Var}}}}[{\left|\phi \right\rangle }^{{{{\rm{B}}}}},{H}_{i}].$$

The fact that pure states achieve the maximum on the right-hand side follows from convexity of the QFI. This bound is of course only possible when the *H*_*i*_ are bounded.

Using the same techniques as for ${{{{\mathcal{S}}}}}_{{{{\rm{avg}}}}}$ (Supplementary Note 6), we can take *H*_*i*_ to be a set of *d*^2^ − 1 traceless generators of SU(*d*) satisfying ${{{\rm{Tr}}}}[{H}_{i}{H}_{j}]={\delta }_{i,j}$, and compute $\overline{{F}_{{{{\rm{Q}}}}}^{* }[{{{\bf{H}}}}]}=\overline{{F}_{{{{\rm{Q}}}}}[{\left|\phi \right\rangle \left\langle \phi \right|}^{{{{\rm{B}}}}},{{{\bf{H}}}}]}=4(d-1)$ (which actually holds for any $\left|\phi \right\rangle$). Thus, for this set of **H** in *d* dimensions, the LHS bound is

33 $$\overline{{F}_{{{{\rm{Q}}}}}[{{{\mathcal{A}}}},{{{\bf{H}}}}]}\le 4(d-1).$$

For a pure state *ψ*^AB^,

34 $$\overline{{F}_{{{{\rm{Q}}}}}^{{{{\rm{B}}}}| {{{\rm{A}}}}}[{\psi }^{{{{\rm{AB}}}}},{{{\bf{H}}}}]}	 =\mathop{\sum} _{i}4{{{\rm{Var}}}}[{\rho }^{{{{\rm{B}}}}},{H}_{i}]\\ 	=4(d-1)+4\mathop{\sum} _{i\ne j}{p}_{i}{p}_{j},$$

so that (33) is violated if and only if *ψ*^AB^ is entangled.

## Supplementary information

Supplementary Information

## Data Availability

All relevant data are available from the authors.

## Code availability

Source codes of the plots are available from the authors upon request.
