# Author Correction: Metrological complementarity reveals the Einstein-Podolsky-Rosen paradox

**DOI:** 10.1038/s41467-021-26597-x

**Published:** 2021-11-04

**Authors:** Benjamin Yadin, Matteo Fadel, Manuel Gessner

**Affiliations:** 1 School of Mathematical Sciences and Centre for the Mathematics and Theoretical Physics of Quantum Non-Equilibrium Systems, University of Nottingham, Nottingham, UK; 2 Wolfson College, University of Oxford, Oxford, UK; 3 Department of Physics, University of Basel, Basel, Switzerland; 4 Laboratoire Kastler Brossel, ENS-Université PSL, CNRS, Sorbonne Université, Collège de France, Paris, France

**Keywords:** Quantum information, Quantum metrology

Correction to: *Nature Communications* 10.1038/s41467-021-22353-3, published online 23 April 2021.

The original version of this Article contained errors in inline Equations in the last paragraph of the “Steering of GHZ states” subsection, which incorrectly read:

For a mixture *ρ* = *p|*GHZ_*ϕ*_^*N*+1^〉〈GHZ_*ϕ*_^*N*+1^| + (1*−p*)${\mathbb{1}}$/2^*N*+1^, using the same measurements we obtain *F*_Q_^B|A^ [*ρ, J*_*z*_] ≥ *p*^2^*N*^2^/[*p* +  2(1−*p*)/2^*N*^], 4Var _Q_^B|A^ [*ρ, Jz*] ≤ (1−*p*)*N*/2^*N*^ + *p*(1−*p*)*N*^2^. Whenever *p* ≫ 2^−*N*^, the criterion witnesses steering.

The correct form should read:

For a mixture *ρ* = *p|*GHZ_*ϕ*_^*N*+1^〉〈GHZ_*ϕ*_^*N*+1^| + (1*−p*)${\mathbb{1}}$/2^*N*+1^, using the same measurements we obtain *F*_Q_^B|A^ [*ρ, J*_*z*_] ≥ *p*^2^*N*^2^/[*p* +  2(1−*p*)/2^*N*^], 4Var _Q_^B|A^ [*ρ, Jz*] ≤ (1−*p*)*N* + *p*(1−*p*)*N*^2^. For large *N*, whenever *p* ⪆ 1/√*N*, the criterion witnesses steering.

This has been corrected in the PDF and HTML versions of the Article.

The original version of the Supplementary Information associated with this Article contained errors in the last paragraph of Supplementary Note 2 “GHZ States with white noise”, which incorrectly read:

For a measurement of *σ*_*z*_ by Alice, Bob’s conditional states are easily found to give

$${{{{{{\rm{Var}}}}}}}_{{{{{{\rm{Q}}}}}}}^{{{{{{\rm{B}}}}}}|{{{{{\rm{A}}}}}}}[\rho ,{J}_{z}]\le \frac{(1-p)N}{4d}+\frac{p(1\,-p){N}^{2}}{4},$$

where *d* = 2^*N*^ . With a *σ*_*x*_ measurement, (3) results in

$${F}_{{{{{{\rm{Q}}}}}}}^{{{{{{\rm{B}}}}}}|{{{{{\rm{A}}}}}}}[\rho ,{J}_{z}]\ge \frac{{p}^{2}{N}^{2}}{p+2(1\,-p)/d}.$$

When *p* ≫ 1/*d* = 2^−*N*^, we can neglect the terms involving *d*. Then *F*_Q_^B|A^ [*ρ, J*_*z*_] ⪆ *pN*^2^, Var_Q_^B|A^ [*ρ, J*_*z*_] ⪅ *p*(1−*p*)*N*^2^< *pN*^2^ for *p* < 1.

The correct version reads instead

For a measurement of *σ*_*z*_ by Alice, Bob’s conditional states are easily found to give

$${{{{{{\rm{Var}}}}}}}_{{{{{{\rm{Q}}}}}}}^{{{{{{\rm{B}}}}}}|{{{{{\rm{A}}}}}}}[\rho ,{J}_{z}]\le \frac{(1-p)N}{4}+\frac{p(1-p){N}^{2}}{4},$$

With a *σ*_*x*_ measurement, (3) results in

$${{F}_{{{{{{\rm{Q}}}}}}}}^{{{{{{\rm{B}}}}}}|{{{{{\rm{A}}}}}}}[\rho ,{J}_{z}]\ge \frac{{p}^{2}{N}^{2}}{p+2(1-p)/d},$$

where *d* = 2^*N*^ . When *p* ≫ 1/*d* = 2^−*N*^, we can neglect the term involving *d*. Then *F*_Q_^B|A^ [*ρ, J*_*z*_] ⪆ *pN*^2^, and the difference

$${F}_{{{{{{\rm{Q}}}}}}}^{{{{{{\rm{B}}}}}}|{{{{{\rm{A}}}}}}}[\rho ,{J}_{z}]-4{{{{{{\rm{Var}}}}}}}_{{{{{{\rm{Q}}}}}}}^{{{{{{\rm{B}}}}}}|{{{{{\rm{A}}}}}}}[\rho ,{J}_{z}]\gtrapprox {p}^{2}{N}^{2}-(1-p)N$$

is positive as long as *N* > (1−*p*)/*p*^*2*^. For large *N*, this condition approximates to *p* ⪆ 1/√*N*.

The HTML has been updated to include a corrected version of the Supplementary Information.

## Supplementary information

Updated Supplementary Information

