# Supplementary material for: Metrological complementarity reveals the Einstein-Podolsky-Rosen paradox
Source: Nat Commun. 2021 Apr 23;12:2410. doi: 10.1038/s41467-021-22353-3 (PMC8065158; doi:10.1038/s41467-021-22353-3)
Supplement: Supplementary file 1 — Supplementary Information [file 41467_2021_22353_MOESM1_ESM.pdf]

Supplementary Notes – Metrological complementarity reveals the Einstein-Podolsky-Rosen paradox

Benjamin Yadin, Matteo Fadel, and Manuel Gessner

SUPPLEMENTARY NOTE 1 - OPTIMAL POVMS FOR ASSISTED METROLOGY

Here, we argue that the optimal measurements performed by Alice can always be taken as rank-1 POVMs, for any assemblage defined by a global quantum state. Suppose a rank-r POVM $E^{\mathrm{A}}_{a|X}$ is optimal for the conditional QFI – that is,

$$F^{\mathrm{B|A}}_{\mathrm{Q}}[\rho^{\mathrm{AB}}, H] = \sum_a p(a|X) F_{\mathrm{Q}}[\rho^{\mathrm{B}}_{a|X}, H]. \tag{1}$$

Then we can decompose (for instance, using the spectral decomposition) $E^{\mathrm{A}}_{a|X} = \sum_{i=1}^{r} E^{\mathrm{A}}_{a,i|X}$, where each $E^{\mathrm{A}}_{a,i|X} \geq 0$ is at most rank-1, This defines a new, fine-grained POVM with conditional states $p(a,i|X) = \mathrm{Tr}_{\mathrm{A}}[E^{\mathrm{A}}_{a,i|X}\rho^{\mathrm{AB}}]$. The original conditional states are obtained by averaging over i: $p(a|X)\rho^{\mathrm{B}}_{a|X} = \sum_i \mathrm{Tr}_{\mathrm{A}}[E^{\mathrm{A}}_{a,i|X}\rho^{\mathrm{AB}}] = \sum_i p(a,i|X)\rho^{\mathrm{B}}_{a,i|X}$. Due to convexity of the QFI,

$$\begin{aligned}
\sum_{a,i} p(a,i|X) F_{\mathrm{Q}}[\rho^{\mathrm{B}}_{a,i|X}, H] &= \sum_a p(a|X) \sum_i \frac{p(a,i|X)}{p(a|X)} F_{\mathrm{Q}}[\rho^{\mathrm{B}}_{a_i|X}, H] \\
&\geq \sum_a p(a|X) F_{\mathrm{Q}}\left[\sum_i \frac{p(a,i|X)}{p(a|X)} \rho^{\mathrm{B}}_{a,i|X}, H\right] \\
&= \sum_a p(a|X) F_{\mathrm{Q}}[\rho^{\mathrm{B}}_{a|X}, H] \\
&= F^{\mathrm{B|A}}_{\mathrm{Q}}[\rho^{\mathrm{AB}}, H].
\end{aligned} \tag{2}$$

Thus the fine-grained POVM is also optimal. The same conclusion holds for the quantum conditional variance, instead using concavity of the variance and the fact that the optimal POVM must minimise the average variance.

SUPPLEMENTARY NOTE 2 - GHZ STATES WITH WHITE NOISE

We first observe that, for any pure state ψ mixed with white noise in d dimensions [1],

$$F_{\mathrm{Q}}\left[p\psi + \frac{(1-p)}{d}\mathbb{1}, H\right] = \frac{4p^2}{p + 2(1-p)/d}\mathrm{Var}[\psi, H]. \tag{3}$$

This follows from choosing an eigenbasis $|i\rangle$, $i = 0, \ldots, d-1$ for the mixed state with $|0\rangle = |\psi\rangle$ and expanding in terms of its eigenvalues λ_i: [2]

$$\begin{aligned}
F_{\mathrm{Q}}\left[p\psi + \frac{(1-p)}{d}\mathbb{1}, H\right] &= 4\sum_{i<j} \frac{(\lambda_i - \lambda_j)^2}{\lambda_i + \lambda_j} |\langle i|H|j\rangle|^2 \\
&= 4\sum_{j>0} \frac{p^2}{p + 2(1-p)/d} \langle 0| H |j\rangle\langle j| H |0\rangle \\
&= \frac{4p^2}{p + 2(1-p)/d} \langle\psi| H(I - |\psi\rangle\langle\psi|)H |\psi\rangle \\
&= \frac{4p^2}{p + 2(1-p)/d} \left[\langle\psi|H^2|\psi\rangle - \langle\psi|H|\psi\rangle^2\right].
\end{aligned} \tag{4}$$

For the shared GHZ state $|\mathrm{GHZ}^{N+1}_{\phi}\rangle = \frac{1}{\sqrt{2}}\left(|0\rangle \otimes |0\rangle^{\otimes N} + e^{i\phi}|1\rangle \otimes |1\rangle^{\otimes N}\right)$ mixed with white noise, any projection onto a pure state on Alice's side results in the same conditional state as obtained for the pure case, up to a mixture with the identity on Bob's side. We keep the same measurement choices for any p, although they may not be optimal when $p < 1$.

For a measurement of σ_z by Alice, Bob's conditional states are easily found to give

$$\mathrm{Var}^{\mathrm{B|A}}_{\mathrm{Q}}[\rho, J_z] \leq \frac{(1-p)N}{4} + \frac{p(1-p)N^2}{4}. \tag{5}$$

With a σ_x measurement, (3) results in

$$F^{\mathrm{B|A}}_{\mathrm{Q}}[\rho, J_z] \geq \frac{p^2N^2}{p + 2(1-p)/d}, \tag{6}$$

where $d = 2^N$. When $p \gg 1/d = 2^{-N}$, we can neglect the term involving d. Then $F_Q^{B|A}[\rho, J_z] \gtrapprox pN^2$, and the difference

$$F_Q^{B|A}[\rho, J_z] - 4\mathrm{Var}_Q^{B|A}[\rho, J_z] \gtrapprox p^2N^2 - (1-p)N \tag{7}$$

is positive as long as $N > (1-p)/p^2$. For large N, this condition approximates to $p \gtrapprox 1/\sqrt{N}$.

SUPPLEMENTARY NOTE 3 - HYBRID CAT STATES

Here, we consider an example of a hybrid system where Alice has a qubit and Bob has a single bosonic mode. Consider the bipartite "cat state"

$$|\psi\rangle_{AB} = \frac{1}{\sqrt{2}}\left(|0\rangle_A|\alpha\rangle_B + |1\rangle_A|{-\alpha}\rangle_B\right), \tag{8}$$

where $|\pm\alpha\rangle$ are coherent states and we take $\alpha \geq 0$ and use a quadrature observable $H = x$.

First consider projection by Alice onto a pure state $|\chi\rangle = a|0\rangle + b|1\rangle$, resulting in Bob's conditional state $|\phi\rangle = (a^*|\alpha\rangle + b^*|{-\alpha}\rangle)/\sqrt{2q}$ with probability $q = 1/2 + \Re[ab^*]e^{-2\alpha^2}$. Representing $|\chi\rangle$ in terms of the unit Bloch vector $\mathbf{r} = (x, y, z)^T$, one finds $q = (1 + xe^{-2\alpha^2})/2$ and the expectation values $q\langle\phi|x|\phi\rangle = \alpha z/\sqrt{2}$, $q\langle\phi|x^2|\phi\rangle = \alpha^2 + q/2$, so the variance is

$$q\mathrm{Var}[|\phi\rangle\langle\phi|, x] = \alpha^2\left[1 - \frac{z^2}{2q}\right] + \frac{q}{2}. \tag{9}$$

Now let Alice use an arbitrary rank-1 POVM with elements $E_m = 2k_m|\chi_m\rangle\langle\chi_m|$, $m = 0, 1, \ldots, M$, $k_m \geq 0$ (which can be assumed without loss of generality – see Supplementary Section). In terms of unit Bloch vectors $\mathbf{r}_m$, these can be expressed as

$$E_m = k_m(\mathbb{1} + \mathbf{r}_m \cdot \boldsymbol{\sigma}) \tag{10}$$

The completeness condition $\sum_m E_k = \mathbb{1}$ is then equivalent to

$$\sum_m k_m = 1, \quad \sum_m k_m\mathbf{r}_m = \mathbf{0}. \tag{11}$$

The probability of outcome m is $p_m = 2k_mq_m$, where $q_m = \|\langle\chi_m|_A|\psi\rangle_{AB}\|^2$. Hence the average variance conditional on this POVM is

$$\sum_m p_m\mathrm{Var}[|\phi_m\rangle\langle\phi_m|, x] = \sum_m\left[2k_m\alpha^2\left(1 - \frac{z_m^2}{2q_m}\right) + \frac{p_m}{2}\right] \tag{12}$$

$$= 2\alpha^2\left[1 - \sum_m \frac{k_mz_m^2}{1 + x_me^{-2\alpha^2}}\right] + \frac{1}{2}. \tag{13}$$

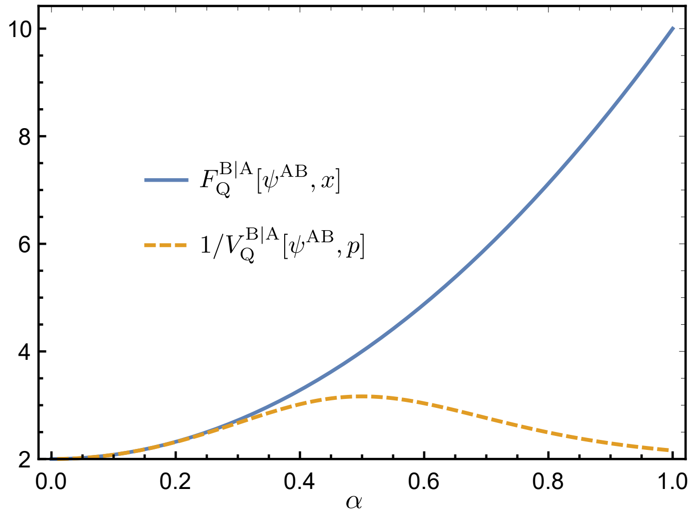

Supplementary Figure 1. **Hybrid cat state.** Alice has a qubit and Bob has a bosonic mode, entangled as in Supplementary Eq. (8). Plot comparing the the conditional QFI $F_Q^{B|A}[\psi^{AB}, x]$ (blue solid) against the lower bound $1/\mathrm{Var}_Q^{B|A}[\psi^{AB}, p]$ (orange dashed), as a function of the coherent state parameter α.

From the fact that $z_m^2/(1+x_m e^{-2\alpha^2}) \in [0,1]$, it follows that $\sum_m k_m z_m^2/(1+x_m e^{-2\alpha^2}) \in [0,1]$, and so

$$\frac{1}{2} \leq \sum_m p_m \mathrm{Var}[|\phi_m\rangle\langle\phi_m|, x] \leq 2\alpha^2 + \frac{1}{2}. \tag{14}$$

The lower bound is saturated by using a measurement in the $\{|0\rangle, |1\rangle\}$ basis, giving

$$\mathrm{Var}_{\mathrm{Q}}^{\mathrm{B|A}}[\psi^{\mathrm{AB}}, x] = \frac{1}{2}. \tag{15}$$

On the other hand, the upper bound is saturated with any POVM whose coordinates z_m all vanish – i.e., all the Bloch vectors lie on the equator – for example the $\{|+\rangle, |-\rangle\}$ basis, giving

$$\frac{1}{4} F_{\mathrm{Q}}^{\mathrm{B|A}}[\psi^{\mathrm{AB}}, x] = 2\alpha^2 + \frac{1}{2}. \tag{16}$$

Hence we see that steering is witnessed via this strategy for any nonzero α, and the violation increases with α.

This may be compared with the Reid criterion using x and p quadrature variances [3]. From Eq. (25) in the main text, a lower bound on the conditional QFI is $F_{\mathrm{Q}}^{\mathrm{B|A}}[\psi^{\mathrm{AB}}, x] \geq 1/\mathrm{Var}_{\mathrm{Q}}^{\mathrm{B|A}}[\psi^{\mathrm{AB}}, p]$. Using the same techniques as above, we find $\mathrm{Var}_{\mathrm{Q}}^{\mathrm{B|A}}[\psi^{\mathrm{AB}}, p] = 1/2 - 2\alpha^2 e^{-4\alpha^2}$, obtained using a measurement in the basis $(|0\rangle \pm i|1\rangle)/\sqrt{2}$. While steering is witnessed for all nonzero α, the violation is greatest at $\alpha = 1/2$ and the criterion becomes less effective as α increases – see Supplementary Fig. 1.

SUPPLEMENTARY NOTE 4 - ATOMIC SPLIT DICKE STATES

In this Section, we apply our criterion to detect steering between two addressable atomic ensembles with a fixed number of total excitations. We first consider in A the deterministic distribution of N atoms in two modes with a fixed number of excitations, as proposed in the main text of our manuscript. Then, in B we analyse a Dicke state that is sent onto a spatial beam splitter to separate each of its mode in two, as was done experimentally with an ensemble of $N = 5\,000$ atoms in Ref. [4].

A. Dicke states with fixed splitting $N_{\mathrm{A}} : N_{\mathrm{B}}$

Consider N atoms split into two addressable modes A and B, with respectively N_{A} and $N_{\mathrm{B}} = N - N_{\mathrm{A}}$ particles. Assume that we know that the internal spin degree of freedom of a total number of $0 \leq k \leq N$ atoms is excited (e.g., from a collective measurement), but we do not know the distribution of the excited atoms into the two modes. The system is described by the split Dicke state

$$|\mathrm{SD}_{k,N_{\mathrm{A}}:N_{\mathrm{B}}}\rangle = \mathcal{N} \sum_{\substack{k_{\mathrm{A}},k_{\mathrm{B}} \\ k_{\mathrm{A}}+k_{\mathrm{B}}=k}} |k_{\mathrm{A}}\rangle \otimes |k_{\mathrm{B}}\rangle \,, \tag{17}$$

where we introduced the eigenstates with k_X excitations of the N_X-particle spin observable J_z^X, for $X = \mathrm{A}, \mathrm{B}$,

$$J_z^X |k_X\rangle = (k_X - N_X/2)|k_X\rangle \,. \tag{18}$$

The range of k_{A} and k_{B} in the sum depends on the values of k, N_{A} and N_{B}. We can formulate constaints, e.g., in terms of k_{A}: Since (i) if there are $k > N_{\mathrm{B}}$ excitations in total, the number of excitations in A must be at least $k_{\mathrm{A}} = k - N_{\mathrm{B}}$ and (ii) N_{A} atoms can show at most N_{A} excitations. These constraints can be taken into account explicitly as

$$|\mathrm{SD}_{k,N_{\mathrm{A}}:N_{\mathrm{B}}}\rangle = \frac{1}{\sqrt{k_{\max} - k_{\min} + 1}} \sum_{k_{\mathrm{A}}=k_{\min}}^{k_{\max}} |k_{\mathrm{A}}\rangle \otimes |k - k_{\mathrm{A}}\rangle \,, \tag{19}$$

where

$$\begin{aligned} k_{\min} &= \max\{0, k - N_{\mathrm{B}}\} \\ k_{\max} &= \min\{k, N_{\mathrm{A}}\} \,. \end{aligned} \tag{20}$$

1. Alice measures J_z^{A}, Bob measures J_z^{A}

To determine the conditional variance, we consider the projection of Alice's system (A) onto eigenstates $|k_{\mathrm{A}}\rangle$ of J_z^{A}. Alice obtains any of the results $k_{\mathrm{A}} = k_{\min}, \ldots, k_{\max}$ with probability $p(k_{\mathrm{A}}|J_z^{\mathrm{A}}) = 1/(k_{\max} - k_{\min} + 1)$, while other results have probability zero. Bob's conditional state $|\Psi_{k_{\mathrm{A}}|J_z^{\mathrm{A}}}\rangle = |k - k_{\mathrm{A}}\rangle$ has zero variance for J_z^{B}, and we obtain

$$\mathrm{Var}_{\mathrm{Q}}^{\mathrm{B|A}}[|\mathrm{SD}_{k,N_{\mathrm{A}}:N_{\mathrm{B}}}\rangle, J_z^{\mathrm{B}}] = \sum_{k_{\mathrm{A}}} p(k_{\mathrm{A}}|J_z^{\mathrm{A}})\mathrm{Var}[|k - k_{\mathrm{A}}\rangle, J_z^{\mathrm{B}}] = 0\ . \tag{21}$$

This measurement is therefore optimal in the sense that it achieves the minimum in the definition of $\mathrm{Var}_{\mathrm{Q}}^{\mathrm{B|A}}[|\mathrm{SD}_{k,N_{\mathrm{A}}:N_{\mathrm{B}}}\rangle, J_z^{\mathrm{B}}]$ [see Eq. (2) in the main text]. This result can be understood intuitively: knowing the total number k of excitations along with the fact that k_{A} of them are found in Alice's subsystem, allows us to predict with certainty that Bob will measure $k_{\mathrm{B}} = k - k_{\mathrm{A}}$ excitations.

2. Alice measures J_x^{A}, Bob estimates θ

For the estimation of a phase shift θ generated by J_z^{B} on Bob's subsystem, let us now consider the measurement of J_x^{A} by Alice, described by projection onto the eigenstates $|k_{\mathrm{A}}\rangle_x = e^{-i\frac{\pi}{2}J_y^{\mathrm{A}}}|k_{\mathrm{A}}\rangle$. The assemblage is given by

$$\begin{aligned}\mathcal{A}(k_{\mathrm{A}}, J_x^{\mathrm{A}}) &= \mathrm{Tr}_{\mathrm{A}}\{(|k_{\mathrm{A}}\rangle_x\langle k_{\mathrm{A}}|_x \otimes \mathbb{1})|\mathrm{SD}_{k,N_{\mathrm{A}}:N_{\mathrm{B}}}\rangle\langle \mathrm{SD}_{k,N_{\mathrm{A}}:N_{\mathrm{B}}}|\} \\ &= p(k_{\mathrm{A}}|J_x^{\mathrm{A}})|\Psi_{k_{\mathrm{A}}|J_x^{\mathrm{A}}}\rangle\langle\Psi_{k_{\mathrm{A}}|J_x^{\mathrm{A}}}|,\end{aligned} \tag{22}$$

with conditional states

$$|\Psi_{k_{\mathrm{A}}|J_x^{\mathrm{A}}}\rangle = \frac{1}{\sqrt{\sum_{k'_{\mathrm{A}}=k_{\min}}^{k_{\max}} |\langle k_{\mathrm{A}}|e^{i\frac{\pi}{2}J_y^{\mathrm{A}}}|k'_{\mathrm{A}}\rangle|^2}} \sum_{k'_{\mathrm{A}}=k_{\min}}^{k_{\max}} \langle k_{\mathrm{A}}|e^{i\frac{\pi}{2}J_y^{\mathrm{A}}}|k'_{\mathrm{A}}\rangle|k - k'_{\mathrm{A}}\rangle, \tag{23}$$

and probabilities

$$p(k_{\mathrm{A}}|J_x^{\mathrm{A}}) = \frac{1}{k_{\max} - k_{\min} + 1} \sum_{k'_{\mathrm{A}}=k_{\min}}^{k_{\max}} |\langle k_{\mathrm{A}}|e^{i\frac{\pi}{2}J_y^{\mathrm{A}}}|k'_{\mathrm{A}}\rangle|^2. \tag{24}$$

The overlap between eigenstates of J_z^{A} and J_x^{A} can be computed using the expression

$$\langle k_{\mathrm{A}}|e^{-i\phi J_y^{\mathrm{A}}}|k'_{\mathrm{A}}\rangle = \sqrt{k'_{\mathrm{A}}!(N_{\mathrm{A}} - k'_{\mathrm{A}})!k_{\mathrm{A}}!(N_{\mathrm{A}} - k_{\mathrm{A}})!} \sum_{n=\max[k'_{\mathrm{A}}-k_{\mathrm{A}},0]}^{\min[k'_{\mathrm{A}},N_{\mathrm{A}}-k_{\mathrm{A}}]} \frac{(-1)^n \cos(\phi/2)^{k_{\mathrm{A}}-k'_{\mathrm{A}}+N_{\mathrm{A}}-2n} \sin(\phi/2)^{2n+k'_{\mathrm{A}}-k_{\mathrm{A}}}}{(k_{\mathrm{A}} - n)!(N_{\mathrm{A}} - k'_{\mathrm{A}} - n)!n!(k'_{\mathrm{A}} - k_{\mathrm{A}} + n)!}\ . \tag{25}$$

We obtain the first and second moments of the conditional states

$$\begin{aligned}\langle J_z^{\mathrm{B}}\rangle_{k_{\mathrm{A}}|J_x^{\mathrm{A}}} &= \frac{1}{\sum_{k'_{\mathrm{A}}=k_{\min}}^{k_{\max}} |\langle k_{\mathrm{A}}|e^{i\frac{\pi}{2}J_y^{\mathrm{A}}}|k'_{\mathrm{A}}\rangle|^2} \sum_{k'_{\mathrm{A}}=k_{\min}}^{k_{\max}} |\langle k_{\mathrm{A}}|\, e^{i\frac{\pi}{2}J_y^{\mathrm{A}}}\, |k'_{\mathrm{A}}\rangle\,|^2\, \langle k - k'_{\mathrm{A}}|\, J_z^{\mathrm{B}}\, |k - k'_{\mathrm{A}}\rangle \\ &= \frac{1}{\sum_{k'_{\mathrm{A}}=k_{\min}}^{k_{\max}} |\langle k_{\mathrm{A}}|e^{i\frac{\pi}{2}J_y^{\mathrm{A}}}|k'_{\mathrm{A}}\rangle|^2} \sum_{k'_{\mathrm{A}}=k_{\min}}^{k_{\max}} |\langle k_{\mathrm{A}}|\, e^{i\frac{\pi}{2}J_y^{\mathrm{A}}}\, |k'_{\mathrm{A}}\rangle\,|^2 \left(k - k'_{\mathrm{A}} - \frac{N_{\mathrm{B}}}{2}\right)\end{aligned} \tag{26}$$

and

$$\langle (J_z^{\mathrm{B}})^2\rangle_{k_{\mathrm{A}}|J_x^{\mathrm{A}}} = \frac{1}{\sum_{k'_{\mathrm{A}}=k_{\min}}^{k_{\max}} |\langle k_{\mathrm{A}}|e^{i\frac{\pi}{2}J_y^{\mathrm{A}}}|k'_{\mathrm{A}}\rangle|^2} \sum_{k'_{\mathrm{A}}=k_{\min}}^{k_{\max}} |\langle k_{\mathrm{A}}|\, e^{i\frac{\pi}{2}J_y^{\mathrm{A}}}\, |k'_{\mathrm{A}}\rangle\,|^2 \left(k - k'_{\mathrm{A}} - \frac{N_{\mathrm{B}}}{2}\right)^2. \tag{27}$$

Since the conditional states are pure, this yields a quantum Fisher information of

$$F_{\mathrm{Q}}[|\Psi_{k_{\mathrm{A}}|J_x^{\mathrm{A}}}\rangle, J_z^{\mathrm{B}}] = 4\mathrm{Var}[|\Psi_{k_{\mathrm{A}}|J_x^{\mathrm{A}}}\rangle, J_z^{\mathrm{B}}] = 4(\langle (J_z^{\mathrm{B}})^2\rangle_{k_{\mathrm{A}}|J_x^{\mathrm{A}}} - \langle J_z^{\mathrm{B}}\rangle^2_{k_{\mathrm{A}}|J_x^{\mathrm{A}}}). \tag{28}$$

Generally, any choice of Alice's measurement setting X yields a lower bound for the quantum conditional Fisher information:

$$F^{\mathrm{B|A}}[\mathcal{A}, X, H] := \sum_a p(a|X) F_{\mathrm{Q}}[\rho^{\mathrm{B}}_{a|X}, H] \leq \max_X F^{\mathrm{B|A}}[\mathcal{A}, X, H] = F_{\mathrm{Q}}^{\mathrm{B|A}}[\mathcal{A}, H]. \tag{29}$$

We obtain the conditional Fisher information

$$F^{\mathrm{B|A}}[|\mathrm{SD}_{k,N_{\mathrm{A}}:N_{\mathrm{B}}}\rangle, J_x^{\mathrm{A}}, J_z^{\mathrm{B}}] = 4 \sum_{k_{\mathrm{A}}=0}^{N_{\mathrm{A}}} p(k_{\mathrm{A}}|J_x^{\mathrm{A}})(\langle (J_z^{\mathrm{B}})^2\rangle_{k_{\mathrm{A}}|J_x^{\mathrm{A}}} - \langle J_z^{\mathrm{B}}\rangle^2_{k_{\mathrm{A}}|J_x^{\mathrm{A}}}). \tag{30}$$

3. *Reduced quantum Fisher information and variance*

The properties of Bob's reduced state provide upper and lower limits on the quantum conditional variance and quantum conditional Fisher information, respectively; cf. Eq. (11) in the main text. Bob's reduced density matrix is given as

$$\rho^{\mathrm{B}} = \frac{1}{k_{\max} - k_{\min} + 1} \sum_{k_{\mathrm{A}}=k_{\min}}^{k_{\max}} |k - k_{\mathrm{A}}\rangle\langle k - k_{\mathrm{A}}|. \tag{31}$$

Let us first calculate the variance of J_z^{B}. The first moment reads

$$\begin{aligned}\langle J_z^{\mathrm{B}}\rangle_{\rho^{\mathrm{B}}} &= \frac{1}{k_{\max} - k_{\min} + 1} \sum_{k_{\mathrm{A}}=k_{\min}}^{k_{\max}} \langle k - k_{\mathrm{A}}|\, J_z^{\mathrm{B}}\, |k - k_{\mathrm{A}}\rangle \\ &= \frac{1}{k_{\max} - k_{\min} + 1} \sum_{k_{\mathrm{A}}=k_{\min}}^{k_{\max}} \left(k - k_{\mathrm{A}} - \frac{N_{\mathrm{B}}}{2}\right) \\ &= \frac{1}{2}\left(2k - N_{\mathrm{B}} - k_{\min} - k_{\max}\right)\end{aligned} \tag{32}$$

and for the second moments, we obtain

$$\begin{aligned}\langle (J_z^{\mathrm{B}})^2\rangle_{\rho^{\mathrm{B}}} &= \frac{1}{k_{\max} - k_{\min} + 1} \sum_{k_{\mathrm{A}}=k_{\min}}^{k_{\max}} \left(k - k_{\mathrm{A}} - \frac{N_{\mathrm{B}}}{2}\right)^2 \\ &= \frac{1}{12}\Big(3(N_{\mathrm{B}} - 2k)^2 + 4k_{\min}^2 + 2k_{\max}(3N_{\mathrm{B}} - 6k + 2k_{\max} + 1) + 2k_{\min}(3N_{\mathrm{B}} - 6k + 2k_{\max} - 1)\Big)\end{aligned} \tag{33}$$

We obtain

$$\mathrm{Var}[\rho^{\mathrm{B}}, J_z^{\mathrm{B}}] = \frac{1}{12}(k_{\max} - k_{\min} + 2)(k_{\max} - k_{\min}). \tag{34}$$

Since the state ρ^{B} is invariant under transformations generated by J_z^{B}, *i.e.* $[\rho^{\mathrm{B}}, J_z^{\mathrm{B}}] = 0$, we obtain that the quantum Fisher information of Bob's reduced state vanishes, $F_{\mathrm{Q}}[\rho^{\mathrm{B}}, J_z^{\mathrm{B}}] = 0$. This can be confirmed explicitly using the expression

$$F_{\mathrm{Q}}[\rho^{\mathrm{B}}, J_z^{\mathrm{B}}] = 2 \sum_{i,j} \frac{(p_i - p_j)^2}{p_i + p_j} |\langle \psi_i | J_z^{\mathrm{B}} | \psi_j\rangle|^2, \tag{35}$$

where $\rho^{\mathrm{B}} = \sum_i p_i |\psi_i\rangle\langle\psi_i|$ is the spectral decomposition of ρ^{B} with eigenvalues $p_i = 1/(k_{\max} - k_{\min} + 1)$ and eigenvectors $|\psi_i\rangle = |k - i\rangle$.

4. *Results for a twin Fock state divided into $N_{\mathrm{A}} = N_{\mathrm{B}} = N/2$*

When the initial state is a twin Fock state, *i.e.* $k = N/2$, that is split in two equal parts with $N_{\mathrm{A}} = N_{\mathrm{B}} = N/2$, the above expressions simplifies further. First of all, we obtain $k_{\min} = 0$, $k_{\max} = N/2$, and $\sum_{k'_{\mathrm{A}}=k_{\min}}^{k_{\max}} |\langle k_{\mathrm{A}}| e^{i\frac{\pi}{2}J_y^{\mathrm{A}}} |k'_{\mathrm{A}}\rangle|^2 = 1$ (as the sum runs over the full basis of the $N_{\mathrm{A}} = N/2$ particle state), giving for the probabilities $p(k_{\mathrm{A}}|J_x^{\mathrm{A}}) = 2/(N+2)$. To simplify the conditional

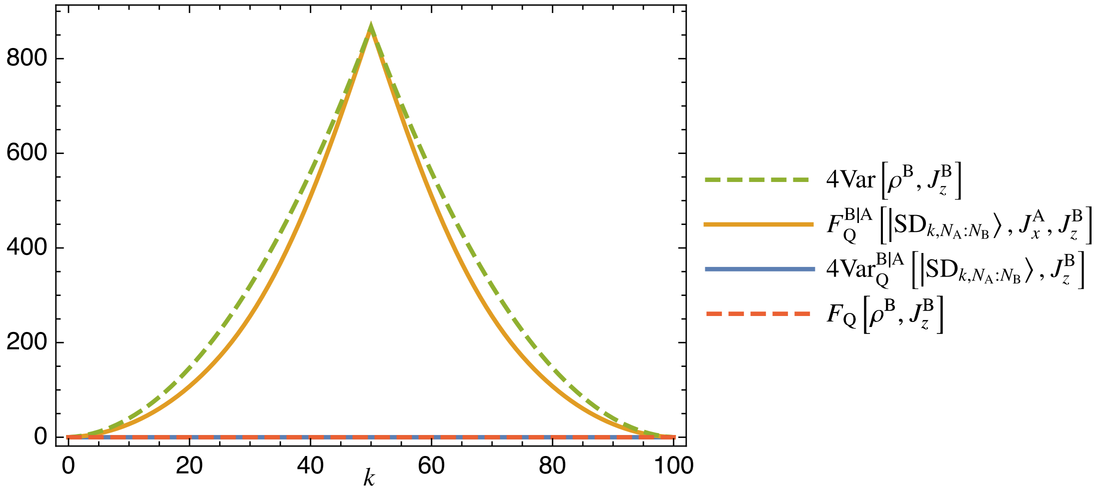

Supplementary Figure 2. **Split Dicke state without partition noise.** Dicke state with $N = 100$ particles and k excitations, deterministically split into $N_{\mathrm{A}} = N_{\mathrm{B}} = N/2$. Plot of Supplementary Eq. (21) (blue), Eq. (30) (yellow), Eq. (34) (green dashed) and Eq. (35) (red dashed).

states (23), note from (25) that $\langle k_{\mathrm{A}}|e^{-iJ_y^{\mathrm{A}}\phi}|k'_{\mathrm{A}}\rangle = \langle N_{\mathrm{A}} - k_{\mathrm{A}}|e^{-iJ_y^{\mathrm{A}}\phi}|N_{\mathrm{A}} - k'_{\mathrm{A}}\rangle$ and that $\langle k_{\mathrm{A}}|e^{-iJ_y^{\mathrm{A}}\phi}|k'_{\mathrm{A}}\rangle = \langle k'_{\mathrm{A}}|e^{iJ_y^{\mathrm{A}}\phi}|k_{\mathrm{A}}\rangle$. Moreover, the matrix elements of J_y^{A} and J_y^{B} coincide since both operators are of the same length. We use this in Supplementary Eq. (23) to write

$$
\begin{aligned}
|\Psi_{k_{\mathrm{A}}|J_x^{\mathrm{A}}}\rangle &= \sum_{k'_{\mathrm{A}}=0}^{N/2} \langle k_{\mathrm{A}}|e^{i\frac{\pi}{2}J_y^{\mathrm{A}}}|k'_{\mathrm{A}}\rangle |N/2 - k'_{\mathrm{A}}\rangle \\
&= \sum_{k'_{\mathrm{A}}=0}^{N/2} \langle k'_{\mathrm{A}}|e^{-i\frac{\pi}{2}J_y^{\mathrm{A}}}|k_{\mathrm{A}}\rangle |N/2 - k'_{\mathrm{A}}\rangle \\
&= \sum_{k'_{\mathrm{A}}=0}^{N/2} \langle N/2 - k'_{\mathrm{A}}|e^{-i\frac{\pi}{2}J_y^{\mathrm{A}}}|N/2 - k_{\mathrm{A}}\rangle |N/2 - k'_{\mathrm{A}}\rangle \\
&= \sum_{k'_{\mathrm{A}}=0}^{N/2} \langle N/2 - k'_{\mathrm{A}}|e^{-i\frac{\pi}{2}J_y^{\mathrm{B}}}|N/2 - k_{\mathrm{A}}\rangle |N/2 - k'_{\mathrm{A}}\rangle \\
&= \underbrace{\left(\sum_{k'_{\mathrm{A}}=0}^{N/2} |N/2 - k'_{\mathrm{A}}\rangle\langle N/2 - k'_{\mathrm{A}}| \right)}_{\mathbb{1}_{\mathrm{B}}} e^{-i\frac{\pi}{2}J_y^{\mathrm{B}}} |N/2 - k_{\mathrm{A}}\rangle \\
&= |N/2 - k_{\mathrm{A}}\rangle_x.
\end{aligned} \tag{36}
$$

Hence, just like in the early examples by EPR and Bohm[5, 6], the state shows perfect correlations in two non-commuting measurement bases, and we may express (19) as

$$
\begin{aligned}
|\mathrm{SD}_{\frac{N}{2},\frac{N}{2}:\frac{N}{2}}\rangle &= \sqrt{\frac{2}{N+2}} \sum_{k_{\mathrm{A}}=0}^{N/2} |k_{\mathrm{A}}\rangle \otimes |N/2 - k_{\mathrm{A}}\rangle \\
&= \sqrt{\frac{2}{N+2}} \sum_{k_{\mathrm{A}}=0}^{N/2} |k_{\mathrm{A}}\rangle_x \otimes |N/2 - k_{\mathrm{A}}\rangle_x.
\end{aligned} \tag{37}
$$

Using (36), we determine the first and second moments of J_z^{B} for the conditional states to be

$$
\langle J_z^{\mathrm{B}}\rangle_{k_{\mathrm{A}}|J_x^{\mathrm{A}}} = 0, \tag{38}
$$

and

$$
\langle (J_z^{\mathrm{B}})^2\rangle_{k_{\mathrm{A}}|J_x^{\mathrm{A}}} = \frac{1}{8}(2k_{\mathrm{A}}(N - 2k_{\mathrm{A}}) + N). \tag{39}
$$

This leads to a quantum conditional Fisher information of

$$F_{\mathrm{Q}}^{\mathrm{B|A}}[|\mathrm{SD}_{\frac{N}{2},\frac{N}{2}:\frac{N}{2}}\rangle, J_z^{\mathrm{B}}] = 4\sum_{k_{\mathrm{A}}=0}^{N/2} p(k_{\mathrm{A}}|J_x^{\mathrm{A}})(\langle (J_z^{\mathrm{B}})^2\rangle_{k_{\mathrm{A}}|J_x^{\mathrm{A}}} = \sum_{k_{\mathrm{A}}=0}^{N/2} \frac{1}{N+2}(2k_{\mathrm{A}}(N-2k_{\mathrm{A}})+N) = \frac{1}{12}N(4+N). \tag{40}$$

By comparison with Supplementary Eq. (34), which yields $\mathrm{Var}[\rho^{\mathrm{B}}, J_z^{\mathrm{B}}] = \frac{1}{48}N(4+N)$, we notice that the upper bound $F_{\mathrm{Q}}^{\mathrm{B|A}}[|\mathrm{SD}_{k,N_{\mathrm{A}}:N_{\mathrm{B}}}\rangle, J_z^{\mathrm{B}}] = 4\mathrm{Var}[\rho^{\mathrm{B}}, J_z^{\mathrm{B}}]$ [see Eq. (11) in the main text] is indeed saturated by this choice of measurement. This shows that no other measurement by Alice could yield a higher average sensitivity on Bob's side. The measurement of J_x^{A} is optimal for assisted metrology with split twin Fock states as it achieves the maximum in the definition of $F_{\mathrm{Q}}^{\mathrm{B|A}}[|\mathrm{SD}_{\frac{N}{2},\frac{N}{2}:\frac{N}{2}}\rangle, J_z^{\mathrm{B}}]$ [see Eq. (5) in the main text].

B. Splitting a Dicke state into two modes

We now focus on a preparation of split Dicke states by a beam splitter operation. Consider a Dicke state with k excitations in the modes a and b, described as

$$|\mathrm{D}_{k,N}\rangle = \frac{(a^\dagger)^k(b^\dagger)^{N-k}}{\sqrt{k!(N-k)!}}|0\rangle. \tag{41}$$

By sending this state onto a beam splitter with ratio $p : 1-p$, both modes are split by into two modes as

$$\begin{aligned} a^\dagger &= \sqrt{p}a_{\mathrm{A}}^\dagger + \sqrt{1-p}a_{\mathrm{B}}^\dagger, \\ b^\dagger &= \sqrt{p}b_{\mathrm{A}}^\dagger + \sqrt{1-p}b_{\mathrm{B}}^\dagger. \end{aligned} \tag{42}$$

As a consequence of the partition noise, the total number of particles in each mode fluctuates. By expanding the binomials that appear upon inserting (42) into (41), this state can be written as

$$\begin{aligned} |\mathrm{SD}_{k,N,p}\rangle &= \sum_{k_{\mathrm{A}}=0}^{k}\sum_{N_{\mathrm{A}}=k_{\mathrm{A}}}^{N-k+k_{\mathrm{A}}} \sqrt{\binom{k}{k_{\mathrm{A}}}\binom{N-k}{N_{\mathrm{A}}-k_{\mathrm{A}}}}\sqrt{p}^{N_{\mathrm{A}}}\sqrt{1-p}^{N-N_{\mathrm{A}}}|k_{\mathrm{A}}\rangle_{N_{\mathrm{A}}}\otimes|k-k_{\mathrm{A}}\rangle_{N-N_{\mathrm{A}}} \\ &= \sum_{N_{\mathrm{A}}=0}^{N}\sum_{k_{\mathrm{A}}=k_{\mathrm{min}}}^{k_{\mathrm{max}}} \sqrt{\binom{k}{k_{\mathrm{A}}}\binom{N-k}{N_{\mathrm{A}}-k_{\mathrm{A}}}}\sqrt{p}^{N_{\mathrm{A}}}\sqrt{1-p}^{N-N_{\mathrm{A}}}|k_{\mathrm{A}}\rangle_{N_{\mathrm{A}}}\otimes|k-k_{\mathrm{A}}\rangle_{N-N_{\mathrm{A}}}, \end{aligned} \tag{43}$$

where $|k_{\mathrm{A}}\rangle_{N_{\mathrm{A}}}$ is an eigenstate of the spin-$N_{\mathrm{A}}/2$ observable J_z^{A}, and similarly for subsystem B.

Alice's measurements of J_z^{A} or J_x^{A}, provide simultaneous information about the spin quantum number and the number of particles N_{A}, whose observable commutes with all spin components. Typically, after a suitable rotation of the state, one measures how many spins point up/down, such that the information about the total number of particles is provided simultaneously. Alice could ignore the information provided by N_{A}, but this coarse-graining would lead to sub-optimal results for the conditional variance and quantum Fisher information; see Supplementary Note .

1. *Alice measures J_z^{A}, Bob measures J_z^{B}*

A measurement of J_z^{A} with the result k_{A} for the magnetic quantum number k_{A} and N_{A} for the number of particles occurs with probability

$$p(k_{\mathrm{A}}, N_{\mathrm{A}}|J_z^{\mathrm{A}}) = \binom{k}{k_{\mathrm{A}}}\binom{N-k}{N_{\mathrm{A}}-k_{\mathrm{A}}}p^{N_{\mathrm{A}}}(1-p)^{N-N_{\mathrm{A}}} \tag{44}$$

for all $k_{\mathrm{min}} \leq k_{\mathrm{A}} \leq k_{\mathrm{max}}$ and with zero probability otherwise. This event produces the conditional state

$$|\mathrm{SD}_{k,N,p}\rangle_{k_{\mathrm{A}},N_{\mathrm{A}}|J_z^{\mathrm{A}}} = |k-k_{\mathrm{A}}\rangle_{N-N_{\mathrm{A}}} \tag{45}$$

on Bob's side. Since these are eigenstates of J_z^{B} we obtain that

$$\mathrm{Var}_{\mathrm{Q}}^{\mathrm{B|A}}[|\mathrm{SD}_{k,N,p}\rangle, J_z^{\mathrm{B}}] = 0\,. \tag{46}$$

This reflects the fact that a measurement of J_z^{A} and N_{A} allows to predict with certainty the measurement results for J_z^{B} and N_{B}.

2. Alice measures J_x^{A}, Bob estimates θ

For the estimation of a phase shift generated by J_z^{A}, we consider measurements of J_x^{A}, together with N_{A}, with results $(k_{\mathrm{A}}, N_{\mathrm{A}})$. A straightforward calculation shows that the event $(k_{\mathrm{A}}, N_{\mathrm{A}})$ occurs with probability

$$p(k_{\mathrm{A}}, N_{\mathrm{A}}|J_x^{\mathrm{A}}) = p^{N_{\mathrm{A}}}(1-p)^{N-N_{\mathrm{A}}} \sum_{k'_{\mathrm{A}}=k_{\min}}^{k_{\max}} \binom{k}{k'_{\mathrm{A}}}\binom{N-k}{N_{\mathrm{A}}-k'_{\mathrm{A}}} |\langle k_{\mathrm{A}}|e^{i\frac{\pi}{2}J_y^{\mathrm{A}}}|k'_{\mathrm{A}}\rangle|^2 \,, \tag{47}$$

for $0 \leq N_{\mathrm{A}} \leq N$ and $0 \leq k_{\mathrm{A}} \leq N_{\mathrm{A}}$. Bob's conditional state in this case reads

$$\begin{aligned}
|\mathrm{SD}_{k,N,p}\rangle_{k_{\mathrm{A}},N_{\mathrm{A}}|J_x^{\mathrm{A}}} &= \frac{1}{\sqrt{p(k_{\mathrm{A}}, N_{\mathrm{A}}|J_x^{\mathrm{A}})}} \sum_{k'_{\mathrm{A}}=k_{\min}}^{k_{\max}} \sqrt{\binom{k}{k'_{\mathrm{A}}}\binom{N-k}{N_{\mathrm{A}}-k'_{\mathrm{A}}}} \sqrt{p}^{N_{\mathrm{A}}} \sqrt{1-p}^{N-N_{\mathrm{A}}} \langle k_{\mathrm{A}}|e^{i\frac{\pi}{2}J_y^{\mathrm{A}}}|k'_{\mathrm{A}}\rangle |k-k'_{\mathrm{A}}\rangle_{N-N_{\mathrm{A}}} \\
&= \frac{1}{\sqrt{\sum_{k'_{\mathrm{A}}=k_{\min}}^{k_{\max}} \binom{k}{k'_{\mathrm{A}}}\binom{N-k}{N_{\mathrm{A}}-k'_{\mathrm{A}}} |\langle k_{\mathrm{A}}|e^{i\frac{\pi}{2}J_y^{\mathrm{A}}}|k'_{\mathrm{A}}\rangle|^2}} \sum_{k'_{\mathrm{A}}=k_{\min}}^{k_{\max}} \sqrt{\binom{k}{k'_{\mathrm{A}}}\binom{N-k}{N_{\mathrm{A}}-k'_{\mathrm{A}}}} \langle k_{\mathrm{A}}|e^{i\frac{\pi}{2}J_y^{\mathrm{A}}}|k'_{\mathrm{A}}\rangle |k-k'_{\mathrm{A}}\rangle_{N-N_{\mathrm{A}}} \,.
\end{aligned} \tag{48}$$

These states have the expectation value

$$\langle J_z^{\mathrm{B}}\rangle_{k_{\mathrm{A}},N_{\mathrm{A}}|J_x^{\mathrm{A}}} = \frac{1}{\sum_{k'_{\mathrm{A}}=k_{\min}}^{k_{\max}} \binom{k}{k'_{\mathrm{A}}}\binom{N-k}{N_{\mathrm{A}}-k'_{\mathrm{A}}} |\langle k_{\mathrm{A}}|e^{i\frac{\pi}{2}J_y^{\mathrm{A}}}|k'_{\mathrm{A}}\rangle|^2} \sum_{k'_{\mathrm{A}}=k_{\min}}^{k_{\max}} \binom{k}{k'_{\mathrm{A}}}\binom{N-k}{N_{\mathrm{A}}-k'_{\mathrm{A}}} |\langle k_{\mathrm{A}}|e^{i\frac{\pi}{2}J_y^{\mathrm{A}}}|k'_{\mathrm{A}}\rangle|^2 \left(k - k'_{\mathrm{A}} - \frac{N_{\mathrm{B}}}{2}\right) \,, \tag{49}$$

and second moment

$$\langle (J_z^{\mathrm{B}})^2\rangle_{k_{\mathrm{A}},N_{\mathrm{A}}|J_x^{\mathrm{A}}} = \frac{1}{\sum_{k'_{\mathrm{A}}=k_{\min}}^{k_{\max}} \binom{k}{k'_{\mathrm{A}}}\binom{N-k}{N_{\mathrm{A}}-k'_{\mathrm{A}}} |\langle k_{\mathrm{A}}|e^{i\frac{\pi}{2}J_y^{\mathrm{A}}}|k'_{\mathrm{A}}\rangle|^2} \sum_{k'_{\mathrm{A}}=k_{\min}}^{k_{\max}} \binom{k}{k'_{\mathrm{A}}}\binom{N-k}{N_{\mathrm{A}}-k'_{\mathrm{A}}} |\langle k_{\mathrm{A}}|e^{i\frac{\pi}{2}J_y^{\mathrm{A}}}|k'_{\mathrm{A}}\rangle|^2 \left(k - k'_{\mathrm{A}} - \frac{N_{\mathrm{B}}}{2}\right)^2 \,, \tag{50}$$

yielding the quantum Fisher information

$$F_{\mathrm{Q}}[|\mathrm{SD}_{k,N,p}\rangle_{k_{\mathrm{A}},N_{\mathrm{A}}|J_x^{\mathrm{A}}}, J_z^{\mathrm{B}}] = 4\mathrm{Var}[|\mathrm{SD}_{k,N,p}\rangle_{k_{\mathrm{A}},N_{\mathrm{A}}|J_x^{\mathrm{A}}}, J_z^{\mathrm{B}}] = 4(\langle (J_z^{\mathrm{B}})^2\rangle_{k_{\mathrm{A}},N_{\mathrm{A}}|J_x^{\mathrm{A}}} - \langle J_z^{\mathrm{B}}\rangle^2_{k_{\mathrm{A}},N_{\mathrm{A}}|J_x^{\mathrm{A}}}) \,. \tag{51}$$

This choice of measurements leads to the conditional Fisher information [see Supplementary Eq. (29)]:

$$F^{\mathrm{B|A}}[|\mathrm{SD}_{k,N,p}\rangle, J_x^{\mathrm{A}}, J_z^{\mathrm{B}}] = \sum_{k_{\mathrm{A}}=0}^{k} \sum_{N_{\mathrm{A}}=k_{\mathrm{A}}}^{N-k+k_{\mathrm{A}}} p(k_{\mathrm{A}}, N_{\mathrm{A}}|J_x^{\mathrm{A}}) F_{\mathrm{Q}}[|\mathrm{SD}_{k,N,p}\rangle_{k_{\mathrm{A}},N_{\mathrm{A}}|J_x^{\mathrm{A}}}, J_z^{\mathrm{B}}] \,. \tag{52}$$

3. Reduced quantum Fisher information and variance

Bob's reduced state is given by

$$\rho^{\mathrm{B}} = \mathrm{Tr}_{\mathrm{A}}\{|\mathrm{SD}_{k,N,p}\rangle\langle \mathrm{SD}_{k,N,p}|\} \tag{53}$$

$$= \sum_{k_{\mathrm{A}}=0}^{k} \sum_{N_{\mathrm{A}}=k_{\mathrm{A}}}^{N-k+k_{\mathrm{A}}} \binom{k}{k_{\mathrm{A}}}\binom{N-k}{N_{\mathrm{A}}-k_{\mathrm{A}}} p^{N_{\mathrm{A}}}(1-p)^{N-N_{\mathrm{A}}} |k-k_{\mathrm{A}}\rangle_{N-N_{\mathrm{A}}} \langle k-k_{\mathrm{A}}|_{N-N_{\mathrm{A}}} \,. \tag{54}$$

It yields the average value

$$\begin{aligned}
\langle J_z^{\mathrm{B}}\rangle_{\rho^{\mathrm{B}}} &= \sum_{k_{\mathrm{A}}=0}^{k} \sum_{N_{\mathrm{A}}=k_{\mathrm{A}}}^{N-k+k_{\mathrm{A}}} \binom{k}{k_{\mathrm{A}}}\binom{N-k}{N_{\mathrm{A}}-k_{\mathrm{A}}} p^{N_{\mathrm{A}}}(1-p)^{N-N_{\mathrm{A}}} \langle k-k_{\mathrm{A}}|J_z^{\mathrm{B}}|k-k_{\mathrm{A}}\rangle_{N-N_{\mathrm{A}}} \\
&= \sum_{k_{\mathrm{A}}=0}^{k} \sum_{N_{\mathrm{A}}=k_{\mathrm{A}}}^{N-k+k_{\mathrm{A}}} \binom{k}{k_{\mathrm{A}}}\binom{N-k}{N_{\mathrm{A}}-k_{\mathrm{A}}} p^{N_{\mathrm{A}}}(1-p)^{N-N_{\mathrm{A}}} \left(k - k_{\mathrm{A}} - \frac{N-N_{\mathrm{A}}}{2}\right) \\
&= \left(\frac{N}{2} - k\right)(1-p).
\end{aligned} \tag{55}$$

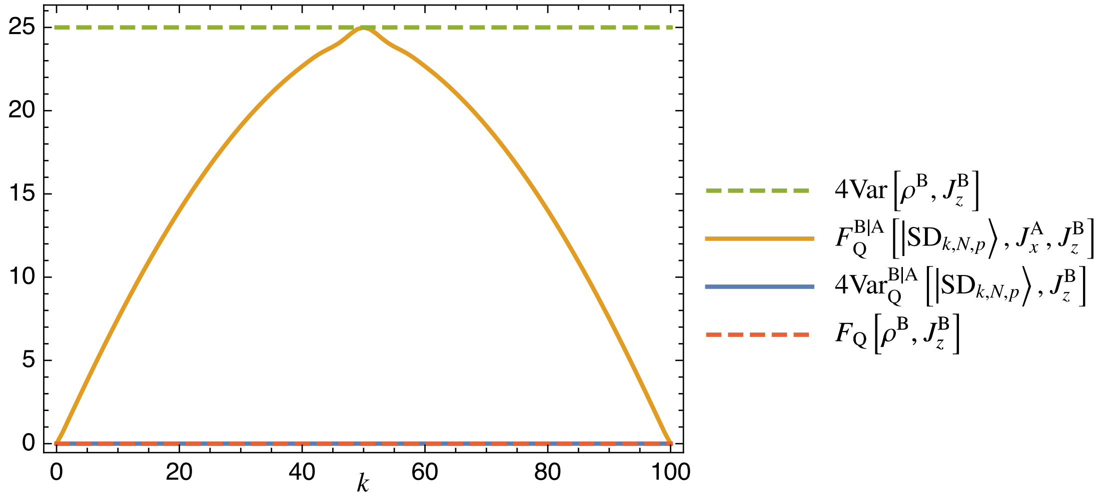

Supplementary Figure 3. **Dicke state split with partition noise.** Dicke state with $N = 100$ particles and k excitations, split into two modes with 50 : 50 ratio. Plot of Supplementary Eq. (46) (blue), Eq. (52) (yellow), Eq. (57) (green dashed) and Eq. (58) (red dashed).

For the second moment, we obtain

$$\langle (J_z^{\rm B})^2\rangle_{\rho^{\rm B}} = \sum_{k_{\rm A}=0}^{k} \sum_{N_{\rm A}=k_{\rm A}}^{N-k+k_{\rm A}} \binom{k}{k_{\rm A}}\binom{N-k}{N_{\rm A}-k_{\rm A}} p^{N_{\rm A}}(1-p)^{N-N_{\rm A}} \left(k-k_{\rm A}-\frac{N-N_{\rm A}}{2}\right)^2$$
$$= \left(\frac{N}{2}-k\right)^2 (1-p)^2 + \frac{N}{4}p(1-p), \tag{56}$$

and the variance reads

$$\text{Var}[\rho^{\rm B}, J_z^{\rm B}] = \frac{N}{4}p(1-p). \tag{57}$$

Since the state is again diagonal in the eigenbasis of $J_z^{\rm B}$, we obtain

$$F_{\rm Q}[\rho^{\rm B}, J_z^{\rm B}] = 0. \tag{58}$$

The data shown in Supplementary Fig. 3 shows that the measurement of $S_x^{\rm A}$ is again optimal for a split twin Fock state, as the conditional Fisher information (52) reaches its upper bound (57).

SUPPLEMENTARY NOTE 5 - BIPARTITE PURE STATES

C. Witnessing with a fixed H

For a shared pure state we can use the saturation of the inequalities labeled by (*) in (11), to express the condition (6) for LHS models as $4\text{Var}[\rho^{\rm B}, H] \leq F_{\rm Q}[\rho^{\rm B}, H]$, whereas in general, this inequality holds in reverse. Hence, steering in this scenario is revealed whenever $4\text{Var}[\rho^{\rm B}, H]$ and $F_{\rm Q}[\rho^{\rm B}, H]$ do not coincide. Even for a fixed H (*i.e.* without optimisation), this condition is close to being a faithful witness of steering: it is satisfied precisely when H is constant on the support of $\rho^{\rm B}$. This follows from Lemma 1 below.

Lemma 1. *$F_{\rm Q}[\rho, H] = 4\text{Var}[\rho, H]$ if and only if $\Pi_\rho H \Pi_\rho \propto \Pi_\rho$, where Π_ρ is the projector on the support of ρ.*

Proof. Using the spectral decomposition $\rho = \sum_i p_i |i\rangle\langle i|$ and with $H_{ij} := \langle i|H|j\rangle$, we can express [7]

$$\text{Var}[\rho, H] - \frac{1}{4}F_{\rm Q}[\rho, H] = 2\sum_{i\neq j} \frac{p_i p_j}{p_i + p_j}|H_{ij}|^2 + \left[\sum_i p_i H_{ii}^2 - \left(\sum_i p_i H_{ii}\right)^2\right], \tag{59}$$

where the second bracketed term, and the terms in the first sum, are all non-negative. When this quantity vanishes, we therefore see that the off-diagonals $H_{ij} = 0$ whenever $p_i, p_j \neq 0$. In addition the bracketed term must vanish, and this is simply the variance

of the diagonals H_{ii} in the distribution p_i. This variance vanishes if and only if the H_{ii} are constant over the range of i such that $p_i \neq 0$. Since

$$\Pi_\rho = \sum_{i:\, p_i \neq 0} |i\rangle\langle i| \,,$$
$$\Pi_\rho H \Pi_\rho = \sum_{\substack{i,j:\\ p_i,p_j \neq 0}} H_{ij}\, |i\rangle\langle j| \,, \tag{60}$$

these conditions can be equivalently expressed neatly as $\Pi_\rho H \Pi_\rho \propto \Pi_\rho$. □

As will be shown in Supplementary Note D, varying over H can make this witness faithful. When Bob's system is a qubit ($d = 2$), without loss of generality we can take the observable to be a Pauli matrix: $H = \mathbf{n} \cdot \boldsymbol{\sigma}$, and then

$$F_{\mathrm{Q}}^{\mathrm{B|A}}[\psi^{\mathrm{AB}}, \mathbf{n} \cdot \boldsymbol{\sigma}] - 4\mathrm{Var}_{\mathrm{Q}}^{\mathrm{B|A}}[\psi^{\mathrm{AB}}, \mathbf{n} \cdot \boldsymbol{\sigma}] = 8\left(1 - \mathrm{Tr}[(\rho^{\mathrm{B}})^2]\right), \tag{61}$$

which is a function of the purity of ρ^{B} and notably independent of the direction $\mathbf{n}$.

D. Optimal measurements

In the case of an overall pure state, it is also possible to determine the optimal measurements needed on Alice's side:

Theorem 1. *i) For a shared pure state ψ^{AB}, an optimal measurement for Alice to achieve $F_{\mathrm{Q}}^{\mathrm{B|A}}[\mathcal{A}, H]$ is*

$$|\tilde{x}_k^*\rangle := \frac{1}{\sqrt{d}} \sum_{l=0}^{d-1} e^{2\pi i k l/d}\, |x_l^*\rangle \,, \tag{62}$$

where $|x_l\rangle$ are the eigenstates of

$$X := \sqrt{\rho^{\mathrm{B}}} H \sqrt{\rho^{\mathrm{B}}} - \langle H \rangle_{\rho^{\mathrm{B}}}\, \rho^{\mathrm{B}}, \tag{63}$$

and $*$ denotes complex conjugation in the Schmidt basis of ψ^{AB}.

ii) Similarly, an optimal measurement to achieve $\mathrm{Var}_{\mathrm{Q}}^{\mathrm{B|A}}[\mathcal{A}, H]$ is $|y_k^*\rangle$, where $|y_k\rangle$ are the eigenstates of the operator

$$Y := \sum_i \frac{2\sqrt{p_i p_j}}{p_i + p_j} H_{ij}\, |i\rangle\langle j| \,. \tag{64}$$

Proof. We follow the proof of Ref. [8], which found a construction for the optimal pure state ensemble in the concave roof of the variance, but stopped short of giving explicit expressions. As shown there, it is sufficient to find a basis in which the diagonals of X vanish. We show that the basis $|\tilde{x}_k\rangle$ (Fourier transformed with respect to the eigenbasis of X) is such a basis. First note that $\mathrm{Tr}\, X = 0$, then writing the spectral decomposition $X = \sum_l x_l\, |x_l\rangle\langle x_l|$,

$$\langle \tilde{x}_k | X | \tilde{x}_k \rangle = \frac{1}{d} \sum_{l,m} e^{2\pi i k(m-l)/d}\, \langle l | X | m \rangle$$
$$= \frac{1}{d} \sum_l x_l = 0. \tag{65}$$

The remainder proceeds as in Ref. [8], which we include for completeness. The optimal ensemble is constructed by $\sqrt{q_k}\, |\psi_k\rangle := \sqrt{\rho}\, |\tilde{x}_k\rangle$ (where we write $\rho^{\mathrm{B}} = \rho$ for brevity). It follows that $q_k = \langle \tilde{x}_k | \rho | \tilde{x}_k \rangle$, and that this is indeed a valid ensemble decomposition for ρ:

$$\sum_k q_k\, |\psi_k\rangle\langle\psi_k| = \sum_k \sqrt{\rho}\, |\tilde{x}_k\rangle\langle\tilde{x}_k|\, \sqrt{\rho} = \rho. \tag{66}$$

Now we have

$$0 = \langle \tilde{x}_k | X | \tilde{x}_k \rangle = \langle \tilde{x}_k | \left(\sqrt{\rho} H \sqrt{\rho} - \langle H \rangle_\rho\, \rho \right)$$
$$= q_k\, \langle \psi_k | H | \psi_k \rangle - \langle H \rangle_\rho\, \langle \tilde{x}_k | \rho | \tilde{x}_k \rangle \,, \tag{67}$$

so $\langle\psi_k|H|\psi_k\rangle = \langle H\rangle_\rho$ whenever $q_k \neq 0$. Thus

$$\sum_k q_k \mathrm{Var}[\psi_k, H] = \sum_k q_k \langle H^2\rangle_{\psi_k} - \sum_k q_k \langle H\rangle^2_{\psi_k}$$
$$= \langle H^2\rangle_\rho - \langle H\rangle^2_\rho = \mathrm{Var}[\rho, H], \tag{68}$$

thus providing the concave roof of the variance.

The measurement basis for Alice to steer Bob into this ensemble follows straightforwardly. Using the Schmidt decomposition $|\psi\rangle_{\mathrm{AB}} = \sum_i \sqrt{p_i}\,|i\rangle\,|i\rangle$, the measurement basis for Alice is $|\tilde{x}_k^*\rangle$, where $*$ denotes complex conjugation in the Schmidt basis. This is seen from

$$\begin{aligned}\langle\tilde{x}_k^*|_{\mathrm{A}}|\psi\rangle_{\mathrm{AB}} &= \sum_i \sqrt{p_i}\,|i\rangle\,\langle\tilde{x}_k|i\rangle^* \\ &= \sum_i \sqrt{p_i}\,|i\rangle\,\langle i|\tilde{x}_k\rangle \\ &= \sqrt{\rho}\,|\tilde{x}_k\rangle \\ &= \sqrt{q_k}\,|\psi_k\rangle\,. \end{aligned}\tag{69}$$

The corresponding statement for $\mathrm{Var}_{\mathrm{Q}}^{\mathrm{B|A}}$ is similarly given by the optimal convex roof ensemble found in Ref. [8], namely $\sqrt{q_k}\,|\psi_k\rangle = \sqrt{\rho}\,|y_k\rangle$. Exactly as above, the measurement required to steer into this ensemble is given by complex conjugation in the Schmidt basis. □

SUPPLEMENTARY NOTE 6 - MAXIMAL AND AVERAGE VIOLATION

Here, we define two quantities involving variation over the generator, namely the maximal and the average violation of the main inequality:

$$\mathcal{S}_{\max}(\mathcal{A}) = \max_{\substack{H:\\ \mathrm{Tr}[H^2]=1}} \left[\frac{1}{4}F_{\mathrm{Q}}^{\mathrm{B|A}}[\mathcal{A}, H] - \mathrm{Var}_{\mathrm{Q}}^{\mathrm{B|A}}[\mathcal{A}, H]\right]^+, \tag{70}$$

$$\mathcal{S}_{\mathrm{avg}}(\mathcal{A}) = (d^2-1)\left[\int \mu(\mathrm{d}\mathbf{n})\frac{1}{4}F_{\mathrm{Q}}^{\mathrm{B|A}}[\mathcal{A}, \mathbf{n}\cdot\mathbf{H}] - \mathrm{Var}_{\mathrm{Q}}^{\mathrm{B|A}}[\mathcal{A}, \mathbf{n}\cdot\mathbf{H}]\right]^+, \tag{71}$$

where $[\cdot]^+ = \max\{0, \cdot\}$ denotes the positive part, and the H_i provide a basis of $\mathrm{SU}(d)$ generators satisfying $\mathrm{Tr}[H_i] = 0$, $\mathrm{Tr}[H_i H_j] = \delta_{i,j}$, and μ is the uniform measure over the sphere of unit vectors $|\mathbf{n}| = 1$.

Note that both quantities are invariant under unitaries on Bob's side. This is immediately evident for $\mathcal{S}_{\max}$. To see it for $\mathcal{S}_{\mathrm{avg}}$, we express the action of some U on the generators as $U^\dagger H_i U = \sum_j R_{ij} H_j$. This action preserves the Hilbert-Schmidt inner product between generators, from which it is found that R must be an orthogonal matrix. Thus $U^\dagger(\mathbf{n}\cdot\mathbf{H})U = (R^T\mathbf{n})\cdot\mathbf{H}$. Using

$$\begin{aligned}F_{\mathrm{Q}}^{\mathrm{B|A}}[U^{\mathrm{B}}\mathcal{A}U^{\mathrm{B}\dagger}, \mathbf{n}\cdot\mathbf{H}] &= F_{\mathrm{Q}}^{\mathrm{B|A}}[\mathcal{A}, U^\dagger(\mathbf{n}\cdot\mathbf{H})U] \\ &= F_{\mathrm{Q}}^{\mathrm{B|A}}[\mathcal{A}, (R^T\mathbf{n})\cdot\mathbf{H}],\end{aligned}\tag{72}$$

and similarly for $\mathrm{Var}_{\mathrm{Q}}^{\mathrm{B|A}}$, it follows that the integral over $\mathbf{n}$ is invariant.

We now compute both quantities for a joint pure state ψ^{AB}.

Theorem 2. *For a joint pure state ψ^{AB}, we have*

$$\mathcal{S}_{\max}(\psi^{\mathrm{AB}}) = \lambda_{\max}[\mathrm{diag}(\mathbf{p}) - \mathbf{p}\mathbf{p}^T], \tag{73}$$

$$\mathcal{S}_{\mathrm{avg}}(\psi^{\mathrm{AB}}) = \sum_{i\neq j} p_i p_j\left(1 + \frac{2}{p_i + p_j}\right), \tag{74}$$

where p_i are the eigenvalues of ρ^{B} and $\lambda_{\max}[M]$ is the largest eigenvalue of a given matrix M.

Proof. From the Methods section, we have,

$$\frac{1}{4}F_{\mathrm{Q}}^{\mathrm{B|A}}[\psi^{\mathrm{AB}},H]-\mathrm{Var}_{\mathrm{Q}}^{\mathrm{B|A}}[\psi^{\mathrm{AB}},H]=\mathrm{Var}[\rho^{\mathrm{B}},H]-\frac{1}{4}F_{\mathrm{Q}}[\rho^{\mathrm{B}},H]. \tag{75}$$

This quantity has been studied by Tóth [7], who shows that

$$\mathrm{Var}[\rho,H]-\frac{1}{4}F_{\mathrm{Q}}[\rho,H]=2\sum_{i\neq j}\frac{p_ip_j}{p_i+p_j}|H_{ij}|^2+\left[\sum_i p_iH_{ii}^2-\left(\sum_i p_iH_{ii}\right)^2\right], \tag{76}$$

and computes the average needed for $\mathcal{S}_{\mathrm{avg}}$.

For $\mathcal{S}_{\max}$, we turn (76) into a matrix expression. We encode the components H_{ij} into a vector whose first d components are the diagonals and remaining $d(d-1)/2$ components are the off-diagonals:

$$\mathbf{v}=(H_{11},H_{22},\ldots,\sqrt{2}H_{12},\sqrt{2}H_{13},\ldots) \tag{77}$$

such that $|\mathbf{v}|^2=\sum_{ij}|H_{ij}|^2=\mathrm{Tr}[H^2]=1$. Similarly define a matrix $M=M_D\oplus M_O$ split into diagonal and off-diagonal parts:

$$[M_D]_{i,j}=\delta_{i,j}p_i-p_ip_j, \tag{78}$$

$$[M_O]_{(ij),(kl)}=\delta_{i,k}\delta_{j,l}\left(\frac{2p_ip_j}{p_i+p_j}\right). \tag{79}$$

We then see that

$$\begin{aligned}\mathrm{Var}[\rho,H]-\frac{1}{4}F_{\mathrm{Q}}[\rho,H]&=\mathbf{v}^{\dagger}M\mathbf{v}\\ \Rightarrow \mathcal{S}_{\max}(\psi^{\mathrm{AB}})&=\lambda_{\max}[M].\end{aligned} \tag{80}$$

Finally, we will show that $\lambda_{\max}[M]=\lambda_{\max}[M_D]\geq\lambda_{\max}[M_O]$. Note that the diagonals of M_O are the harmonic means of each pair of p_i,p_j, $(i\neq j)$, and so

$$\lambda_{\max}[M_O]=\frac{2p_1p_2}{p_1+p_2}=:m_*, \tag{81}$$

where without loss of generality we have ordered $p_1\geq p_2\geq\cdots\geq p_d$. Note that $m_*\geq p_2$.

From the Weyl inequalities on eigenvalues [9, Theorem III.2.1], we can bound

$$\begin{aligned}\lambda_{\max}[M_D]&\leq\lambda_{\max}[\mathrm{diag}(\mathbf{p})]+\lambda_{\max}[-\mathbf{p}\mathbf{p}^T]\\&=p_1,\end{aligned} \tag{82}$$

$$\begin{aligned}\lambda_{\max}[M_D]&\geq\lambda_2[\mathrm{diag}(\mathbf{p})]+\lambda_{d-1}[-\mathbf{p}\mathbf{p}^T]\\&=p_2,\end{aligned} \tag{83}$$

where λ_k denotes the kth largest eigenvalue. So we have $p_2\leq\lambda_{\max}[M_D]\leq p_1$ – we will now obtain a stronger lower bound. Let us inspect the eigenvector condition $M_D\mathbf{w}=m\mathbf{w}$:

$$\begin{aligned}p_iw_i-p_i\sum_j p_jw_j&=mw_i\quad\forall i\\ \Rightarrow w_i=\frac{p_i\bar{w}}{p_i-m},&\ \bar{w}=\sum_j p_jw_j.\end{aligned} \tag{84}$$

This determines the eigenvector corresponding to the eigenvalue m. Multiplying by p_i and summing over i,

$$\begin{aligned}\bar{w}&=\sum_i\frac{p_i^2\bar{w}}{p_i-m}\\ \Rightarrow\bar{w}&=0\text{ or }\sum_i\frac{p_i^2}{p_i-m}=1.\end{aligned} \tag{85}$$

Now $\bar{w} \neq 0$ or else we would have $\mathbf{w} = \mathbf{0}$ by (84). Therefore m satisfies $g(m) := \sum_i p_i^2/(p_i - m) = 1$. The function g is strictly increasing in regions where it is continuous:

$$g'(m) = \sum_i \frac{p_i^2}{(p_i - m)^2} > 0. \tag{86}$$

Evaluating $g(m_*)$ from (81),

$$\begin{aligned} g(m_*) = &\frac{p_1^2}{p_1 - 2p_1p_2/(p_1+p_2)} + \frac{p_2^2}{p_2 - 2p_1p_2/(p_1+p_2)} \\ &+ \sum_{i>2} \frac{p_i^2}{p_i - m_*} \\ &= (p_1+p_2)\left(\frac{p_1}{p_1-p_2} + \frac{p_2}{p_2-p_1}\right) + \sum_{i>2}\frac{p_i^2}{p_i - m_*} \\ &= p_1 + p_2 + \sum_{i>2}\frac{p_i^2}{p_i - m_*} \\ &\le p_1 + p_2 \\ &\le 1, \end{aligned} \tag{87}$$

since $m_* \ge p_2 \ge p_3 \ge \dots$. We are working in the region $p_2 \le m \le p_1$ as shown above, in which g is continuous and thus strictly increasing. So in order to have $g(m) = 1$, it must be that $m \ge m_*$. In other words, $\lambda_{\max}[M_D] \ge \lambda_{\max}[M_O]$. □

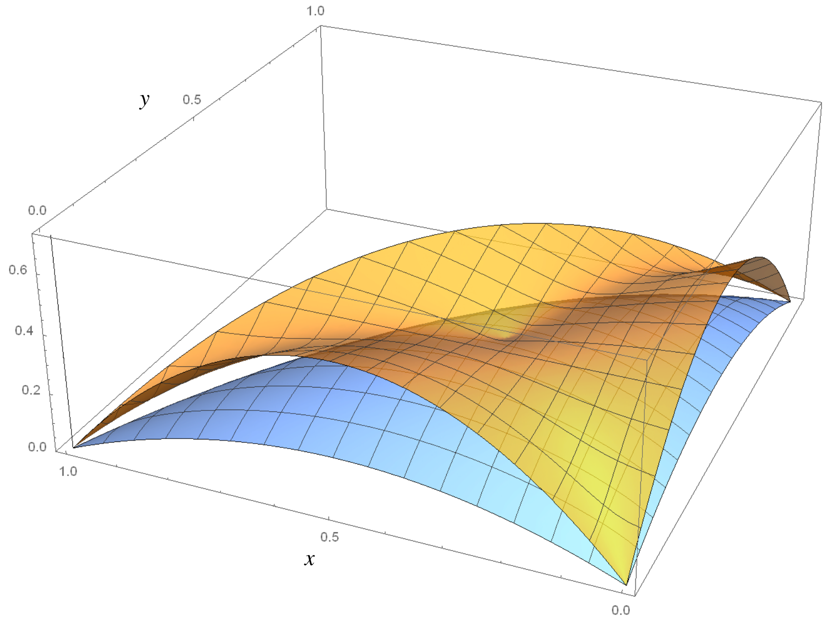

Supplementary Figure 4. **Steering quantifiers for pure bipartite states.** A plot of the quantities $\mathcal{S}_{\text{avg}}/(d^2-1)$ (blue) and $\mathcal{S}_{\max}$ (orange) for a pure state with Schmidt coefficients x, y, $1-x-y$. Apart from the extreme points, using this normalisation they coincide at $x = y = 1/3$, corresponding to the maximally entangled state with $d = 3$.

For pure states, steering is equivalent to entanglement, therefore any steering measure must reduce to an entanglement measure when evaluated on pure states [10, 11].

Corollary 1. *$\mathcal{S}_{\text{avg}}$ is a full entanglement measure for pure states. $\mathcal{S}_{\max}$ is a faithful witness of entanglement for pure states, but not a monotone under LOCC.*

Proof. It is easy to see that $\mathcal{S}_{\text{avg}}$ vanishes if and only if $p_ip_j = 0\ \forall i \neq j$, *i.e.* $p_1 = 1$, $p_2 = p_3 = \dots = 0$, meaning that ρ^{B} is pure.

A function $f(\rho^{\mathrm{B}})$ satisfies strong monotonicity under LOCC if and only if (i) f is a symmetric function of the eigenvalues of ρ^{B}, (ii) f is expansible (meaning that zero eigenvalues can be appended without changing the value) and (iii) f is concave [12, 13]. Properties (i) and (ii) are evident from the expression (74), while (iii) follows from concavity of V and convexity of F_{Q}.

$\mathcal{S}_{\max}$ vanishes if and only if ψ^{AB} is separable, and it is a symmetric, expansible function of $\mathbf{p}$. However, it is not an entanglement monotone, since its maximal value is attained for $\mathbf{p} = (1/2, 1/2, 0, \dots)$ (observed numerically; see Supplementary Fig. 4), which in $d > 2$ does not correspond to the maximally entangled state. □

For general mixed states, the values in Theorem 2 are upper bounds to $\mathcal{S}_{\mathrm{avg}}$, $\mathcal{S}_{\max}$.

Now we characterise further aspects of these quantities applied to general assemblages. At this stage, it is convenient to introduce the notation

$$\Delta^{\mathrm{B|A}}[\mathcal{A}, H] := \frac{1}{4} F_{\mathrm{Q}}^{\mathrm{B|A}}[\mathcal{A}, H] - \mathrm{Var}_{\mathrm{Q}}^{\mathrm{B|A}}[\mathcal{A}, H]. \tag{88}$$

Lemma 2. *$\mathcal{S}_{\max}$ and $\mathcal{S}_{\mathrm{avg}}$ are convex.*

Proof. We first show that $F_{\mathrm{Q}}^{\mathrm{B|A}}[\mathcal{A}, H]$ is convex in $\mathcal{A}$. By definition, a convex combination of two assemblages takes the form $[q\mathcal{A} + (1-q)\mathcal{A}'](a|X) = q\mathcal{A}(a|X) + (1-q)\mathcal{A}'(a|X) = qp(a|X)\rho_{a|X} + (1-q)p'(a|X)\rho'_{a|X}$. From convexity of the QFI, we see

$$\begin{aligned}
F_{\mathrm{Q}}^{\mathrm{B|A}}[q\mathcal{A} + (1-q)\mathcal{A}', H] &\leq \max_X \left(\sum_a qp(a|X) F_{\mathrm{Q}}[\rho_{a|X}, H] + (1-q)p'(a|X) F_{\mathrm{Q}}[\rho'_{a|X}, H] \right) \\
&\leq q \max_X \sum_a p(a|X) F_{\mathrm{Q}}[\rho_{a|X}, H] + (1-q) \max_X \sum_a p'(a|X) F_{\mathrm{Q}}[\rho'_{a|X}, H] \\
&= q F_{\mathrm{Q}}^{\mathrm{B|A}}[\mathcal{A}, H] + (1-q) F_{\mathrm{Q}}^{\mathrm{B|A}}[\mathcal{A}', H].
\end{aligned} \tag{89}$$

The same argument shows that $\mathrm{Var}_{\mathrm{Q}}^{\mathrm{B|A}}$ is concave in $\mathcal{A}$, and so $\Delta^{\mathrm{B|A}}$ is also convex.

For $\mathcal{S}_{\max}$, we also need to employ convexity of the function $[\cdot]^+$:

$$\begin{aligned}
\mathcal{S}_{\max}(q\mathcal{A} + [1-q]\mathcal{A}') &\leq \max_H \left[q\Delta^{\mathrm{B|A}}[\mathcal{A}, H] + (1-q)\Delta^{\mathrm{B|A}}[\mathcal{A}', H] \right]^+ \\
&\leq \max_H \left(q \left[\Delta^{\mathrm{B|A}}[\mathcal{A}, H] \right]^+ + (1-q) \left[\Delta^{\mathrm{B|A}}[\mathcal{A}', H] \right]^+ \right) \\
&\leq q \max_H \Delta^{\mathrm{B|A}}[\mathcal{A}, H] + (1-q) \max_H \Delta^{\mathrm{B|A}}[\mathcal{A}', H] \\
&= q\mathcal{S}_{\max}(\mathcal{A}) + (1-q)\mathcal{S}_{\max}(\mathcal{A}').
\end{aligned} \tag{90}$$

The reasoning is very similar for $\mathcal{S}_{\mathrm{avg}}$:

$$\begin{aligned}
\mathcal{S}_{\mathrm{avg}}(q\mathcal{A} + [1-q]\mathcal{A}') &\leq (d^2 - 1) \left[\int \mu(\mathrm{d}\mathbf{n})\, q\Delta^{\mathrm{B|A}}[\mathcal{A}, \mathbf{n} \cdot \mathbf{H}] + (1-q)\Delta^{\mathrm{B|A}}[\mathcal{A}', \mathbf{n} \cdot \mathbf{H}] \right]^+ \\
&\leq q(d^2 - 1) \left[\int \mu(\mathrm{d}\mathbf{n})\, \Delta^{\mathrm{B|A}}[\mathcal{A}, \mathbf{n} \cdot \mathbf{H}] \right]^+ + (1-q)(d^2 - 1) \left[\int \mu(\mathrm{d}\mathbf{n})\, \Delta^{\mathrm{B|A}}[\mathcal{A}', \mathbf{n} \cdot \mathbf{H}] \right]^+ \\
&= q\mathcal{S}_{\mathrm{avg}}(\mathcal{A}) + (1-q)\mathcal{S}_{\mathrm{avg}}(\mathcal{A}').
\end{aligned} \tag{91}$$

□

The following results demonstrate an aspect of these quantities that is well behaved: being unchanged whenever Bob appends an additional pure state $\phi = |\phi\rangle\langle\phi|$ to his side. This is a necessary condition for monotonicity under 1W-LOCC, and may be a useful step in proving that, if true.

Lemma 3. *Suppose an ancillary system B' in a pure state ϕ is appended to Bob's side – the new assemblage is denoted $\mathcal{A} \otimes \phi^{\mathrm{B}}$. Then, for any observable H acting on the system BB', we have*

$$\Delta^{\mathrm{BB'|A}}[\mathcal{A} \otimes \phi^{\mathrm{B'}}, H] = \Delta^{\mathrm{B|A}}[\mathcal{A}, \tilde{H}], \tag{92}$$

where $\tilde{H} := \langle\phi|_{B'} H |\phi\rangle_{B'}$.

Proof. Lemma 3 from Ref. [14] says that, for any projector Π with the property $\Pi\rho = \rho$,

$$F_{\mathrm{Q}}[\rho, H] - 4\mathrm{Var}[\rho, H] = F_{\mathrm{Q}}[\rho, \Pi H\Pi] - 4\mathrm{Var}[\rho, \Pi H\Pi]. \tag{93}$$

We take $\rho = \rho_{a|X} \otimes \phi^{\mathrm{B}'}$, $\Pi = I \otimes \phi^{\mathrm{B}'}$, such that $\Pi H\Pi = \tilde{H} \otimes \phi^{\mathrm{B}'}$, and then

$$\begin{aligned} F_{\mathrm{Q}}[\rho_{a|X} \otimes \phi^{\mathrm{B}'}, H] &= F_{\mathrm{Q}}[\rho_{a|X} \otimes \phi^{\mathrm{B}'}, \tilde{H} \otimes \phi^{\mathrm{B}'}] + 4\mathrm{Var}[\rho_{a|X} \otimes \phi^{\mathrm{B}'}, H] - 4\mathrm{Var}[\rho_{a|X} \otimes \phi^{\mathrm{B}'}, \tilde{H} \otimes \phi^{\mathrm{B}'}] \\ &= F_{\mathrm{Q}}[\rho_{a|X}, \tilde{H}] + 4\mathrm{Var}[\rho_{a|X} \otimes \phi^{\mathrm{B}'}, H] - 4\mathrm{Var}[\rho_{a|X}, \tilde{H}] \\ &= F_{\mathrm{Q}}[\rho_{a|X}, \tilde{H}] + 4\langle H^2\rangle_{\rho_{a|X}\otimes\phi^{\mathrm{B}'}} - 4\langle H\rangle^2_{\rho_{a|X}\otimes\phi^{\mathrm{B}'}} - 4\langle \tilde{H}^2\rangle_{\rho_{a|X}} + 4\langle \tilde{H}\rangle^2_{\rho_{a|X}} \\ &= F_{\mathrm{Q}}[\rho_{a|X}, \tilde{H}] + 4\langle\langle\phi|_{B'} H^2|\phi\rangle_{B'} - \tilde{H}^2\rangle_{\rho_{a|X}}, \end{aligned} \tag{94}$$

resulting in

$$\begin{aligned} F_{\mathrm{Q}}^{\mathrm{B|A}}[\mathcal{A} \otimes \phi^{\mathrm{B}'}, H] &= \max_X \sum_a p(a|X)\left(F_{\mathrm{Q}}[\rho_{a|X}, \tilde{H}] + 4\langle\langle\phi|_{B'} H^2|\phi\rangle_{B'} - \tilde{H}^2\rangle_{\rho_{a|X}}\right) \\ &= \max_X \sum_a p(a|X) F_{\mathrm{Q}}[\rho_{a|X}, \tilde{H}] + 4\langle\langle\phi|_{B'} H^2|\phi\rangle_{B'} - \tilde{H}^2\rangle_{\rho^{\mathrm{B}}} \\ &= F_{\mathrm{Q}}^{\mathrm{B|A}}[\mathcal{A}, \tilde{H}] + 4\langle\langle\phi|_{B'} H^2|\phi\rangle_{B'} - \tilde{H}^2\rangle_{\rho^{\mathrm{B}}}. \end{aligned} \tag{95}$$

Similarly,

$$\begin{aligned} \mathrm{Var}_{\mathrm{Q}}^{\mathrm{B|A}}[\mathcal{A} \otimes \phi^{\mathrm{B}'}, H] &= \min_X \sum_a p(a|X)\mathrm{Var}[\rho_{a|X} \otimes \phi^{\mathrm{B}'}, H] \\ &= \min_X \sum_a p(a|X)\left(\langle\langle\phi|_{B'} H^2|\phi\rangle_{B'}\rangle_{\rho_{a|X}} - \langle\tilde{H}\rangle^2_{\rho_{a|X}}\right) \\ &= \min_X \sum_a p(a|X)\left(\langle\langle\phi|_{B'} H^2|\phi\rangle_{B'} - \tilde{H}^2\rangle_{\rho_{a|X}} + \mathrm{Var}[\rho_{a|X}, \tilde{H}]\right) \\ &= \langle\langle\phi|_{B'} H^2|\phi\rangle_{B'} - \tilde{H}^2\rangle_{\rho^{\mathrm{B}}} + \min_X \sum_a p(a|X)\mathrm{Var}[\rho_{a|X}, \tilde{H}] \\ &= \langle\langle\phi|_{B'} H^2|\phi\rangle_{B'} - \tilde{H}^2\rangle_{\rho^{\mathrm{B}}} + \mathrm{Var}_{\mathrm{Q}}^{\mathrm{B|A}}[\mathcal{A}, \tilde{H}]. \end{aligned} \tag{96}$$

Putting these last two equations together gives the claimed result. □

Lemma 4. $\mathcal{S}_{\max}(\mathcal{A} \otimes \phi^{\mathrm{B}'}) = \mathcal{S}_{\max}(\mathcal{A})$

Proof. Take any H on BB′ such that $\mathrm{Tr}[H] = 0$, $\mathrm{Tr}[H^2] = 1$. Picking any product basis such that $|0\rangle_{B'} = |\phi\rangle_{B'}$, we have

$$\begin{aligned} \mathrm{Tr}[H^2] &= \sum_{i,j,k,l} |\langle ik|H|jl\rangle|^2 \\ &\geq \sum_{i,j} |\langle i0|H|j0\rangle|^2 \\ &= \sum_{i,j} |\langle i|\tilde{H}|j\rangle|^2 \\ &= \mathrm{Tr}[\tilde{H}^2]. \end{aligned} \tag{97}$$

Therefore $\mathrm{Tr}[\tilde{H}^2] \leq 1$; however, we need not have $\mathrm{Tr}[\tilde{H}] = 0$. By appropriately shifting and scaling, we have a new generator

$$G := \frac{\tilde{H} - \mathrm{Tr}[\tilde{H}]I}{\mathrm{Tr}[\tilde{H}^2]} \tag{98}$$

satisfying $\mathrm{Tr}[G] = 0$, $\mathrm{Tr}[G^2] = 1$. (This is all assuming $\tilde{H} \neq 0$, otherwise the remainder of the argument is trivial.) From

Lemma 3, picking the optimal H for witnessing steering on ABB',

$$\begin{aligned}\mathcal{S}_{\max}(\mathcal{A}\otimes\phi^{\mathrm{B}'}) &= \frac{1}{4}F_{\mathrm{Q}}^{\mathrm{BB}'|\mathrm{A}}[\mathcal{A}\otimes\phi^{\mathrm{B}'},H] - \mathrm{Var}^{\mathrm{BB}'|\mathrm{A}}[\mathcal{A}\otimes\phi^{\mathrm{B}'},H]\\ &= \frac{1}{4}F_{\mathrm{Q}}^{\mathrm{B|A}}[\mathcal{A},\tilde{H}] - \mathrm{Var}_{\mathrm{Q}}^{\mathrm{B|A}}[\mathcal{A},\tilde{H}]\\ &= \mathrm{Tr}[\tilde{H}^2]\left(\frac{1}{4}F_{\mathrm{Q}}^{\mathrm{B|A}}[\mathcal{A},G] - \mathrm{Var}_{\mathrm{Q}}^{\mathrm{B|A}}[\mathcal{A},G]\right)\\ &\le \frac{1}{4}F_{\mathrm{Q}}^{\mathrm{B|A}}[\mathcal{A},G] - \mathrm{Var}_{\mathrm{Q}}^{\mathrm{B|A}}[\mathcal{A},G]\\ &\le \mathcal{S}_{\max}(\mathcal{A}).\end{aligned} \tag{99}$$

The reverse inequality is easily seen by noting that the set of generators on BB′ includes those of the form $H^{\mathrm{B}}\otimes I^{\mathrm{B}'}$, and so

$$\begin{aligned}\mathcal{S}_{\max}(\mathcal{A}\otimes\phi^{\mathrm{B}'}) &\ge \max_{H^{\mathrm{B}}}\left(\frac{1}{4}F_{\mathrm{Q}}^{\mathrm{BB}'|\mathrm{A}}[\mathcal{A}\otimes\phi^{\mathrm{B}'},H^{\mathrm{B}}\otimes I^{\mathrm{B}'}] - \mathrm{Var}^{\mathrm{BB}'|\mathrm{A}}[\mathcal{A}\otimes\phi^{\mathrm{B}'},H^{\mathrm{B}}\otimes I^{\mathrm{B}'}]\right)\\ &= \max_{H^{\mathrm{B}}}\left(\frac{1}{4}F_{\mathrm{Q}}^{\mathrm{B|A}}[\mathcal{A},H^{\mathrm{B}}] - \mathrm{Var}_{\mathrm{Q}}^{\mathrm{B|A}}[\mathcal{A},H^{\mathrm{B}}]\right)\\ &= \mathcal{S}_{\max}(\mathcal{A}).\end{aligned} \tag{100}$$

□

Lemma 5. $\mathcal{S}_{\mathrm{avg}}(\mathcal{A}\otimes\phi^{\mathrm{B}'}) = \mathcal{S}_{\mathrm{avg}}(\mathcal{A})$

Proof. Let B and B′ have respective dimensions d and d'. B thus has a set $\boldsymbol{h}$ of orthonormal SU(d) generators h_μ, $\mu = 1,\ldots,g := d^2-1$, and similarly B′ has $g' := d'^2 - 1$ generators $\boldsymbol{h}'$. It is convenient to set $h_0 = I/\sqrt{d}$, which completes the orthonormal basis of Hermitian generators. For the joint system BB′, we can choose a set $\boldsymbol{H}$ of $G := (dd')^2 - 1$ generators $H_{\mu,\nu} := h_\mu\otimes h'_\nu$ where $\mu = 0,1,\ldots,g$, $\nu = 0,1,\ldots,g'$, but $\mu=\nu=0$ is excluded (since we are only interested in operators with zero trace).

Applying Lemma 3, we have $\Delta^{\mathrm{BB}'|\mathrm{A}}[\mathcal{A}\otimes\phi^{\mathrm{B}'},\boldsymbol{N}\cdot\boldsymbol{H}] = \Delta^{\mathrm{B|A}}[\mathcal{A},\boldsymbol{N}\cdot\tilde{\boldsymbol{H}}]$, where $\tilde{H}_{\mu,\nu} = \langle\phi|h_\nu|\phi\rangle\, h_\mu$. Then

$$\begin{aligned}\boldsymbol{N}\cdot\tilde{\boldsymbol{H}} &= \sum_{\mu=0}^{g}\sum_{\nu=0}^{g'} N_{\mu,\nu}\,\langle\phi|h_\nu|\phi\rangle\, h_\mu\\ &= \sum_{\mu=1}^{g}\sum_{\nu=1}^{g'} N_{\mu,\nu}\,\langle\phi|h_\nu|\phi\rangle\, h_\mu + \sum_{\mu=1}^{g}\frac{N_{\mu,0}}{\sqrt{d'}}h_\mu + \sum_{\nu=1}^{g'}\frac{\langle\phi|h_\nu|\phi\rangle}{\sqrt{d}}I.\end{aligned} \tag{101}$$

The constant term does not contribute to the QFI or the variance, so

$$\begin{aligned}\Delta^{\mathrm{BB}'|\mathrm{A}}[\mathcal{A}\otimes\phi^{\mathrm{B}'},\boldsymbol{N}\cdot\boldsymbol{H}] &= \Delta^{\mathrm{B|A}}[\mathcal{A},\boldsymbol{n}\cdot\boldsymbol{h}],\\ n_\mu &= \sum_{\nu=0}^{g'}\langle\phi|h_\nu|\phi\rangle\, N_{\mu,\nu}.\end{aligned} \tag{102}$$

In order to perform the integral, we rotate to a convenient choice of coordinates $M_{\mu\nu} = R_{\mu\nu,\lambda\sigma}N_{\lambda\sigma}$. R is chosen to be an orthogonal matrix with the components in the first g rows set to $R_{\mu 0,\lambda\sigma} := \delta_{\mu,\lambda}\langle\phi|h_\sigma|\phi\rangle$. This is possible because of the orthonormality of the vectors $\boldsymbol{v}^\mu := (\delta_{\mu,\lambda}\langle\phi|h_\sigma|\phi\rangle)_{\lambda,\sigma}$ (it is easy to show that $\sum_\sigma\langle\phi|h_\sigma|\phi\rangle^2 = 1$). These coordinates are such that $M_{\mu 0} = n_\mu$.

Letting $A(D)$ denote the surface area of a D-dimensional sphere, we have

$$\begin{aligned}\mathcal{S}_{\mathrm{avg}}(\mathcal{A}\otimes\phi^{\mathrm{B}'}) &= \frac{G}{A(G-1)}\left[\int_{|\boldsymbol{N}|=1}\mathrm{d}\boldsymbol{N}\,\Delta^{\mathrm{BB}'|\mathrm{A}}[\mathcal{A}\otimes\phi^{\mathrm{B}'},\boldsymbol{N}\cdot\boldsymbol{H}]\right]^+\\ &= \frac{G}{A(G-1)}\left[\int_{|\boldsymbol{M}|=1}\mathrm{d}\boldsymbol{M}\,\Delta^{\mathrm{B|A}}[\mathcal{A},\sum_\mu M_{\mu 0}h_\mu]\right]^+.\end{aligned} \tag{103}$$

Now we employ generalised spherical coordinates, where G cartesian coordinates x_i, $i = 1, \ldots, G$ are represented in terms of angles $\theta_1, \ldots, \theta_{G-1}$ (and a radius which here is $r = 1$):

$$
\begin{aligned}
x_1 &= \cos\theta_1, \\
x_2 &= \sin\theta_1 \cos\theta_2, \\
&\vdots \\
x_{G-1} &= \sin\theta_1 \sin\theta_2 \ldots \sin\theta_{G-2} \cos\theta_{G-1}, \\
x_G &= \sin\theta_1 \sin\theta_2 \ldots \sin\theta_{G-2} \sin\theta_{G-1},
\end{aligned} \tag{104}
$$

where all θ_i are in the range $[0, \pi)$ apart from $\theta_{G-1} \in [0, 2\pi)$. The volume element is $\mathrm{d}\boldsymbol{x} = \prod_{i=1}^{G} \sin^{G-i-1}(\theta_i)\mathrm{d}\theta_i$. We set the $M_{\mu 0}$ to equal the last g of these coordinates, i.e., $M_{\mu 0} = x_{G-g+\mu}$.

It is easy to show that $\sum_{\mu=1}^{g} M_{\mu 0}^2 = \sum_{\mu=1}^{g} x_{G-g+\mu}^2 = \prod_{i=1}^{G-g} \sin^2\theta_i$. Thus we can normalise $\sum_\mu M_{\mu 0} h_\mu$ by dividing by this norm, obtaining

$$
\begin{aligned}
\hat{\boldsymbol{n}} \cdot \boldsymbol{h} &:= \frac{1}{\sqrt{\sum_\mu M_{\mu 0}^2}} \sum_\mu M_{\mu 0} h_\mu \\
&= \pm\cos(\theta_{G-g+1}) h_1 \pm \sin(\theta_{G-g+1})\cos(\theta_{G-g+2}) h_2 + \cdots \pm \sin(\theta_{G-g+1}) \ldots \sin(\theta_{G-1}) h_g.
\end{aligned} \tag{105}
$$

From this, we see that $\hat{\boldsymbol{n}}$ varies over all unit vectors in the subspace spanned by the final g cartesian coordinates. By factoring the norm outside the QFI and variance, we have

$$
\begin{aligned}
\mathcal{S}_{\mathrm{avg}}(\mathcal{A} \otimes \phi^{\mathrm{B}'}) &= \frac{G}{A(G-1)} \left[\int \left(\prod_{i=1}^{G} \sin^{G-i-1}(\theta_i) \mathrm{d}\theta_i \right) \left(\prod_{i=1}^{G-g} \sin^2(\theta_i) \right) \Delta^{\mathrm{B|A}}[\mathcal{A}, \hat{\boldsymbol{n}} \cdot \boldsymbol{h}] \right]^+ \\
&= \frac{G}{A(G-1)} \left[\int \left(\prod_{i=1}^{G-g} \sin^{G-i+1}(\theta_i) \mathrm{d}\theta_i \right) \int \left(\prod_{i=G-g+1}^{G} \sin^{G-i-1}(\theta_i) \mathrm{d}\theta_i \right) \Delta^{\mathrm{B|A}}[\mathcal{A}, \hat{\boldsymbol{n}} \cdot \boldsymbol{h}] \right]^+ \\
&= \frac{G}{A(G-1)} \left[\left(\prod_{i=1}^{G-g} \int \sin^{G-i+1}(\theta) \mathrm{d}\theta \right) \frac{A(g-1)}{g} \mathcal{S}_{\mathrm{avg}}(\mathcal{A}) \right]^+ .
\end{aligned} \tag{106}
$$

Now

$$
\begin{aligned}
\int \sin^k(\theta) \mathrm{d}\theta &= \frac{\pi^{1/2} \Gamma\left(\frac{k+1}{2}\right)}{\Gamma\left(\frac{k+2}{2}\right)}, \\
A(g-1) &= \frac{2\pi^{g/2}}{\Gamma\left(\frac{g}{2}\right)},
\end{aligned} \tag{107}
$$

so that

$$
\begin{aligned}
\mathcal{S}_{\mathrm{avg}}(\mathcal{A} \otimes \phi^{\mathrm{B}'}) &= \frac{GA(g-1)}{gA(G-1)} \pi^{(G-g)/2} \frac{\Gamma\left(\frac{G+1}{2}\right)}{\Gamma\left(\frac{G+2}{2}\right)} \cdot \frac{\Gamma\left(\frac{G}{2}\right)}{\Gamma\left(\frac{G+1}{2}\right)} \ldots \frac{\Gamma\left(\frac{g+4}{2}\right)}{\Gamma\left(\frac{g+3}{2}\right)} \cdot \frac{\Gamma\left(\frac{g+2}{2}\right)}{\Gamma\left(\frac{g+3}{2}\right)} \mathcal{S}_{\mathrm{avg}}(\mathcal{A}) \\
&= \frac{2G\pi^{g/2}\Gamma\left(\frac{G}{2}\right)}{2g\pi^{G/2}\Gamma\left(\frac{g}{2}\right)} \pi^{(G-g)/2} \cdot \frac{\Gamma\left(\frac{g}{2}+1\right)}{\Gamma\left(\frac{G}{2}+1\right)} \mathcal{S}_{\mathrm{avg}}(\mathcal{A}) \\
&= \frac{\frac{G}{2}\Gamma\left(\frac{G}{2}\right)}{\frac{g}{2}\Gamma\left(\frac{g}{2}\right)} \cdot \frac{\Gamma\left(\frac{g}{2}+1\right)}{\Gamma\left(\frac{G}{2}+1\right)} \mathcal{S}_{\mathrm{avg}}(\mathcal{A}) \\
&= \mathcal{S}_{\mathrm{avg}}(\mathcal{A}),
\end{aligned} \tag{108}
$$

having used the identity $x\Gamma(x) = \Gamma(x+1)$. □

[1] G. Tóth and I. Apellaniz, Quantum metrology from a quantum information science perspective, J. Phys. A **47**, 424006 (2014).

[2] M. G. A. Paris, Quantum Estimation for Quantum Technology, Int. J. Quant. Inf. **07**, 125–137 (2009).

[3] E. G. Cavalcanti and M. D. Reid, Criteria for generalized macroscopic and mesoscopic quantum coherence, Phys. Rev. A **77**, 062108 (2008)

[4] K. Lange, J. Peise, B. Lücke, I. Kruse, G. Vitagliano, I. Apellaniz, M. Kleinmann, G. Tóth, and C. Klempt, Entanglement between two spatially separated atomic modes, Science **360**, 416–418 (2018).

[5] A. Einstein, B. Podolsky, and N. Rosen, Can Quantum-Mechanical Description of Physical Reality Be Considered Complete? Phys. Rev. **47**, 777 (1935).

[6] M. D. Reid, P. D. Drummond, W. P. Bowen, E. G. Cavalcanti, P. K. Lam, H. A. Bachor, U. L. Andersen, and G. Leuchs, Colloquium: The Einstein-Podolsky-Rosen paradox: From concepts to applications, Rev. Mod. Phys. **81**, 1727 (2009).

[7] G. Tóth, Lower bounds on the quantum Fisher information based on the variance and various types of entropies, arXiv:1701.07461.

[8] S. Yu, Quantum Fisher Information as the Convex Roof of Variance, arXiv:1302.5311.

[9] R. Bhatia, *Matrix Analysis*, Graduate Texts in Mathematics Vol. 169 (Springer New York, New York, 1997)

[10] H. M. Wiseman, S. J. Jones, and A. C. Doherty, Steering, Entanglement, Nonlocality, and the Einstein-Podolsky-Rosen Paradox, Phys. Rev. Lett. **98**, 140402 (2007).

[11] N. Gisin, Bell's inequality holds for all non-product states, Phys. Lett. A, **154**, 201 (1991).

[12] G. Vidal, Entanglement Monotones, J. Mod. Opt. **47**, 355 (2000).

[13] R. Horodecki, P. Horodecki, M. Horodecki, and K. Horodecki, Quantum Entanglement Rev. Mod. Phys. **81**, 865 (2009).

[14] B. Morris, B. Yadin, M. Fadel, T. Zibold, P. Treutlein, and G. Adesso, Entanglement between Identical Particles Is a Useful and Consistent Resource, Phys. Rev. X **10**, 041012 (2020)
